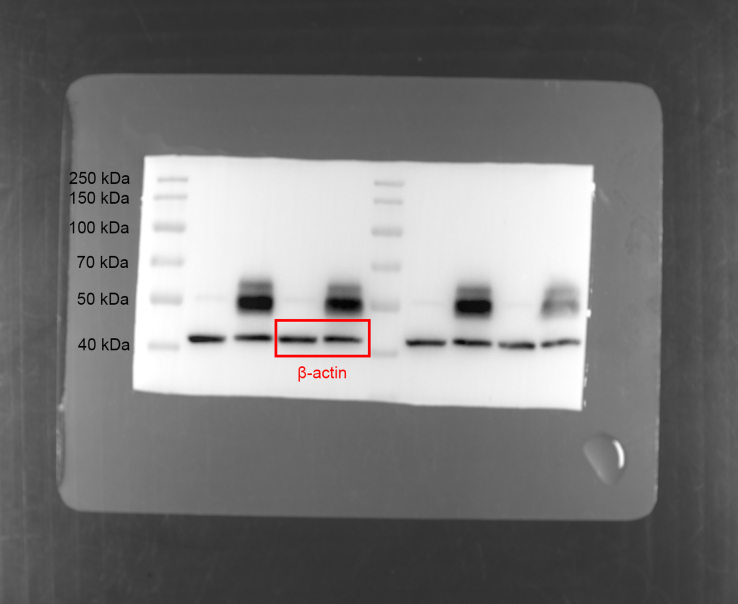# The mechanism of Ca^2+^-independent activation of BKCa channels in mouse inner hair cells and the crucial role of the BK channels in auditory perception

**DOI:** 10.1016/j.jbc.2024.107970

**Published:** 2024-11-07

**Authors:** Zhong-Shan Shen, Jun Gan, Bing Xu, Ya-Lin Chen, Fei-Fei Zhang, Jun-Wei Ji, Dan-Hua Chen, Yuehua Qiao, Qiong-Yao Tang, Zhe Zhang

**Affiliations:** 1 Jiangsu Province Key Laboratory of Anesthesiology, Xuzhou Medical University, Xuzhou, Jiangsu Province, China; 2 Jiangsu Province Key Laboratory of Anesthesia and Analgesia Application Technology, Xuzhou, Medical University, Xuzhou, Jiangsu Province, China; 3 NMPA Key Laboratory for Research and Evaluation of Narcotic and Psychotropic Drugs, Xuzhou Medical University, Xuzhou, Jiangsu Province, China; 4 Otorhinolaryngology Department, The Affiliated Hospital of Xuzhou Medical University, Xuzhou, Jiangsu Province, China; 5 Auditory Engineering Laboratory of Jiangsu Province, Xuzhou Medical University, Xuzhou, Jiangsu Province, China

**Keywords:** the BK channel, mechanical force, special isoforms, LRRC52, inner hair cell, hearing loss

## Abstract

BK channels are expressed in mouse cochlear inner hair cells (IHCs) and exhibit Ca^2+^-independent activation at negative potentials. However, the mechanism underlying Ca^2+^-independent activation of the BK channels in mouse IHCs remains unknown. In this study, we found the BK channel expressed in IHCs contains both the stress-axis regulated exon 2 variant and an alternative splice of exon9 (alt9), which significantly shift the voltage dependence of the BK channels when coexpressed with LRRC52 in 0 [Ca^2+^]_i_. Furthermore, we discovered that mechanical force also induces negative shifts in the voltage dependence of IHC-expressed BK channels. Thus, we propose that the additive effects of mechanical force, special isoforms, and LRRC52 coexpression on voltage dependence shifts may account for the Ca^2+^-independent activation of the BK channel in IHC. Additionally, we found that the IHCs-specific deletion of the BK channels causes hearing damage in mice. Our study suggests a mechanism for Ca^2+^-independent activation in IHCs and highlights the crucial role of the BK channel in auditory perception.

The BK channels are ubiquitously expressed potassium channels that play an essential role in many physiological processes, such as the regulation of blood pressure, neural excitability, and synaptic transmission (1, 2, 3). Dysfunction of BK channels is also implicated in various pathological processes, including epilepsy, cardiovascular disease, and ataxia (4, 5, 6, 7, 8, 9).

The *Slo1* gene encodes the pore-forming α subunit of the BK channel, which assembles into a tetrameric protein complex to form a functional channel (10). The α subunits can also coassemble with β subunits (β1–β4) or γ subunits (γ1–γ4) in a 1:1 ratio to form an octameric channel with distinct biophysical properties, such as inactivation, shifts in voltage dependence, and altered activation and deactivation kinetics (11, 12, 13). The activation of BK channels is regulated by cytosolic Ca^2+^ and changes in membrane potential. The S4 segment of the α subunit of the BK channel contains several positively charged residues that respond to change in membrane voltage, while the long C terminus of the BK channel has two RCK domains that can bind Ca^2+^ (14, 15).

The BK channel also plays an essential role in cochlear inner hair cells (IHCs) (16). Previous research has revealed seven variants located in the cytosolic domain of the BK channel expressed in turtle cochlear hair cells (17). Single-channel recordings of the BK channel in turtle IHCs demonstrate a ∼320 pS unitary conductance and varied kinetics in hair cells tuned to different frequencies (18). These isoforms also exhibit significantly different Ca^2+^ sensitivity and voltage dependency when expressed in *Xenopus* oocytes (18). Subsequently, similar work in chicks and mice led to the identification of nine and 27 BK channel variants, respectively (19, 20). The BK channel splice variants in chicks could differentially distribute in hair cells along the tonotopic axis of the chick cochlea (21). The 27 full-length BK variants in mice result from alternative splicing at X1 to X7 splicing sites; five of these variants of them were cloned from postnatal days 30 mice, while the other 22 variants were cloned from postnatal days 14 mice, indicating a regulation of alternative splicing during cochlea development. However, in rats, only six variants of the BK channel α subunits and two β subunits were equally distributed in the IHCs and did not contribute to tuning (22). Further research has shown the BK activation in the mouse IHCs demonstrated a profound leftward shift of the conductance-voltage (GV) curve (23), a shift to more negative potentials than the shift of the GV curve of the BK channel when coexpressed with the LRRC52 subunit (γ2) in *Xenopus* oocytes (24). However, the mechanism underlying this shift is still unclear.

Among the BK variants, alternative splicing that contained the exon 22 was found to decrease following hypophysectomy, and this exon was named the stress-axis regulated exon (STREX) due to its regulation by hormones. The STREX-1 variant contains a 174-bp exon 22, whereas STREX-2 includes an additional 9-bp exon 21 (Fig. 1*B*) (25). The STREX exon has been identified as a key determinant of the mechanical sensitivity of the BK channel and is expressed in IHCs (19, 26); thus, we propose that the STREX variants contribute to the mechanical sensitivity of the BK channels in IHCs. However, how mechanical energy from sound contributes to the BK activity remains unknown. Furthermore, there is an alternative splice involving exon 9 of the BK channel (e9alt), which is located in the linker between the S6 segment and cytoplasmic domain (27). Some mutations in the linker have been suggested to alter the voltage dependence of the BK channel (28). However, whether the alternative splice exon 9 contributes to the activation shift of the BK channel in the cochlea remains unknown.

**Figure 1:**
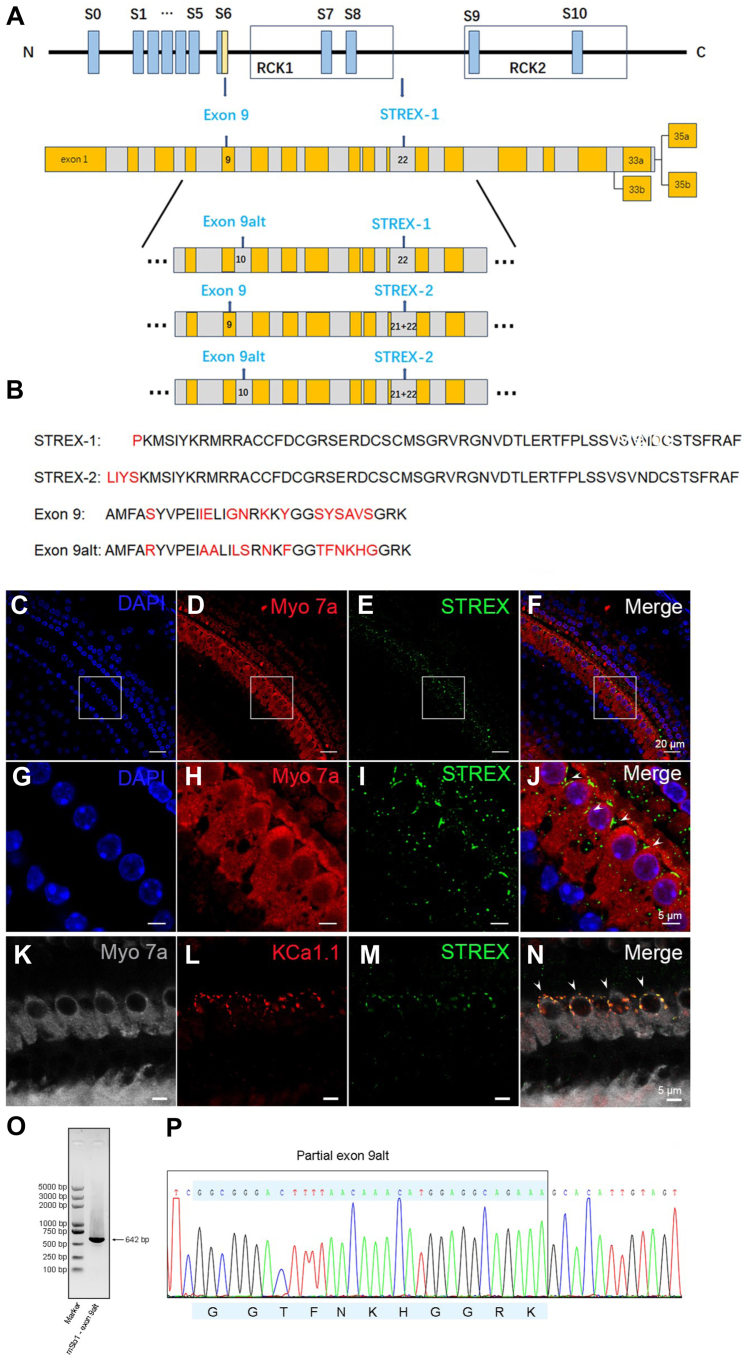
**The STREX and exon 9alt variants of the BK channel in mice IHCs.***A, upper*: schematic view of mSlo1 structural elements: S0-S10 hydrophobic domains; RCK1 and RCK2 regions. The region corresponding to exon 9 or exon 9alt (e9alt) is highlighted in *yellow*. *Lower:* A total of 37 exons are derived. Odd (*yellow*) and even (*gray*) numbered exons are represented across splice sites, where the length of each colored box implies the proportional length of each exon. Variants from up to low: STREX-1, STREX-1-e9alt, STREX-2, and STREX-2-e9alt, respectively. *B,* alignment and amino acid sequence difference of the fragments of STREX-1, STREX-2, exon 9, and exon 9alt, respectively. *C–F,* confocal images of mice cochlea sections confirm that STREX-positive cells (*green*, *E*) express the IHC marker Myosin 7a (*red*, *D*), and the nuclear marker 4′,6-diamidino-2-phenylindole (*blue*, *C*). The coexpression of the STREX and Myosin 7a (*yellow*, *F*) in the same cells of IHC. *G–J,* high magnification images in the area of the square frame, the *white arrows* show the STREX is mostly expressed in the IHC. *K–N,* confocal images of mice cochlea sections confirm the colocalization of KCNMA1 (*red*, *L*) and the STREX (*green*, *M*) in the IHCs (*gray*, *K*). *O,* the gel electrophoresis result of 642 bp mSlo1-exon 9alt PCR product. *P,* chromatographic view of sequencing results for partial mSlo1-exon 9alt DNA product. IHC, inner hair cell; STREX, stress-axis regulated exon.

On the other hand, the contribution of the BK channel to auditory perception also remains unclear. One study using Slo1^−/−^ mice demonstrates progressive high-frequency hearing loss starting at 8 weeks of age in mice (29). Deletion of the BK channel also influences acoustic signal transmission; the response AN fibers in BK^−/−^ mice shows deteriorated precision in spike timing, indicating an increased variance in first spike latency in response to tone bursts (30). However, another study involving the BK channel, β1, and β4 KO mice reported normal auditory brainstem responses (ABRs) thresholds in the BK^−/−^, β1, and β4 mice, while the BK^−/−^ mice were found to be resistant to noise-induced hearing loss (31). But two recent studies attributed hearing loss to the malfunction of the BK channel in the IHCs in their genetically modified mouse models (32, 33). These conflicting results motivate us to further examine the function of BK channels in the mouse cochlea.

In this study, we characterized the biophysical properties of the BK channel STREX variants (including STREX-1 and STREX-2) and exon 9 variants (e9alt) by examining their voltage dependence and mechanical sensitivity across different cytosolic Ca^2+^ concentrations. We also assessed the voltage dependence and mechanical sensitivity of these isoforms when coexpressed with LRRC52. In addition, we generated a mouse model targeting IHCs-specific deletion of the BK channel and tested auditory sensitivity by ABR. We concluded that the additive effects of mechanical forces, specific isoforms, and the coexpression of the LRRC52 may account for the profound voltage dependence shift in the absence of cytosolic Ca^2+^ for the activation of the stretch-activated BK channel in the IHCs and expose the role of the BK channel in auditory perception.

## Results

To investigate whether the BK channels expressed in the mouse IHCs have mechanical sensitivity, we first examined whether the mechanical sensing exon (STREX exon 22) (Fig. 1, *A* and *B*) of the BK channel is translated in the IHCs. To do this, we used an anti-STREX antibody to detect the expression of the STREX exon in the IHCs by immunofluorescent staining. The STREX positive staining signal was located at the neck of the apical cellular membrane of IHCs (Fig. 1, *C–J*), consistent with the previously described location of the BK channel in mouse IHCs (29, 34, 35). Since the STREX exon has an alternative splice variant that has been proven to express in IHCs (19), we cloned this variant and named it STREX-2. The sequence differences are shown in Fig. 1*B*. In addition, we also tested whether the alternative splicing of the S6 segment, which we named exon9alt (e9alt), is expressed in the cochlea (Fig. 1*B* lower). The S6 segment was successfully cloned from the cochlear tissue of mice by RT-PCR and sequenced, with the results showing that exon9alt is also expressed in the cochlea (Fig. 1, *K* and *L*).

**Figure 2:**
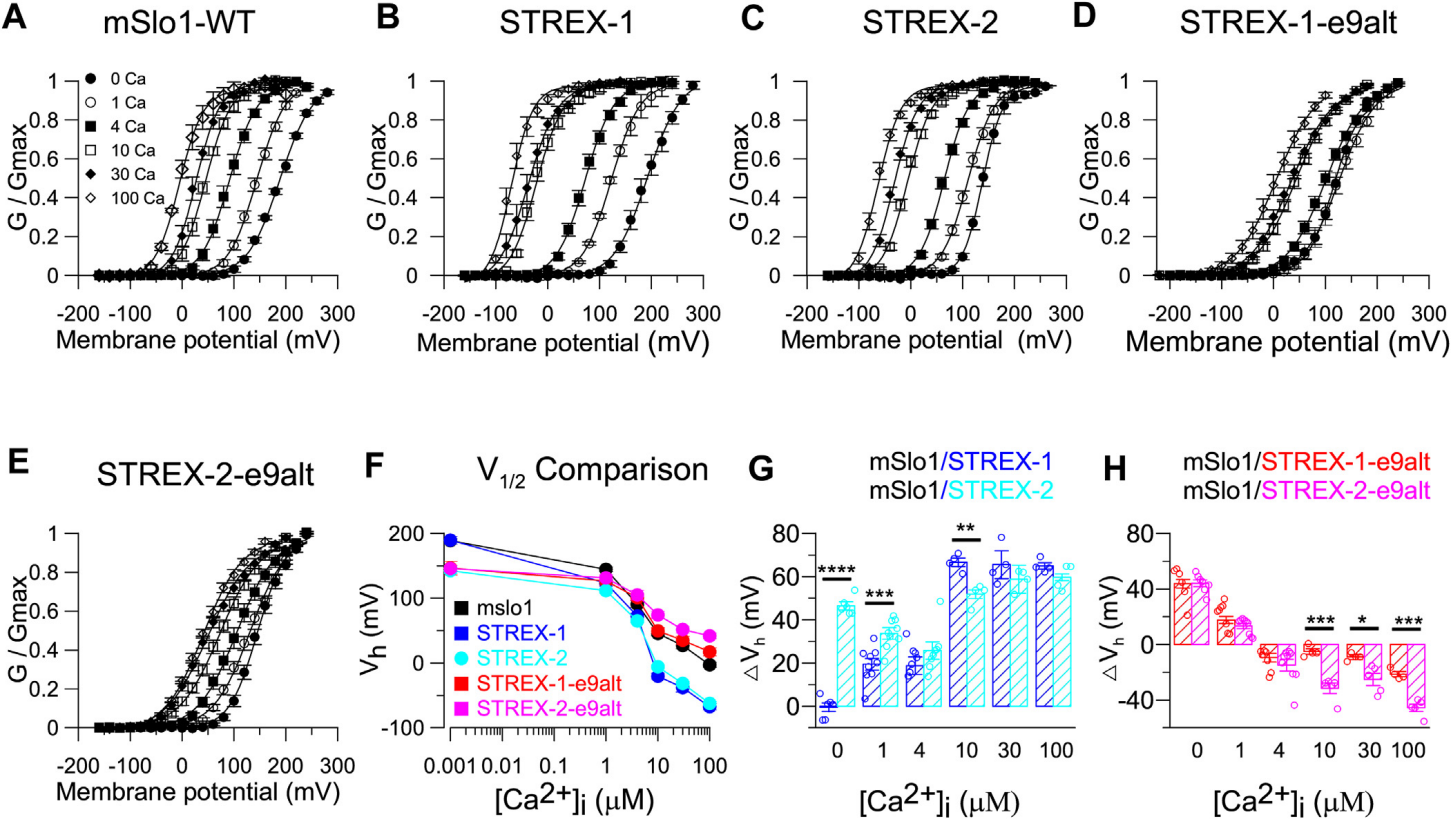
**Effects of STREX and exon 9alt splicing on the activation properties of BK channels.***A–E,* normalized GV curves for mSlo1-WT (*A*), STREX-1 (*B*), STREX-2 (*C*), STREX-1-e9alt (*D*), and STREX-2-e9alt (*E*) channels in 0∼100 μM [Ca^2+^]_i_. All GV relationships are fitted with the Boltzmann equation (*solid line*). *F,* the V_h_-[Ca^2+^]_i_ relationship of mSlo1-WT, STREX-1, STREX-2, STREX-1-e9alt, and STREX-2-e9alt channels. V_h_ is the voltage where the GV relation is at half-maximum. *G,* the ΔV_h_ caused by STREX-1 and STREX-2 exon in 0∼100 μM [Ca^2+^]_i_, respectively. At each [Ca^2+^]_i_, ΔV_h_ = V_h(mSlo1)_ – V_h (STREX-1)_ (*blue*) or ΔV_h_ = V_h(mSlo1)_ – V_h(STREX-2)_ (*cyan*). The data are presented as mean ± SE, n = 4 to 9 patches. *H,* the ΔV_h_ of the BK channel caused by STREX-1-e9alt and STREX-2-e9alt in 0∼100 μM [Ca^2+^]_i_, respectively. At each [Ca^2+^]_i_, ΔV_h_ = V_h(mSlo1)_ – V_h(STREX-1-e9alt)_ (*red*) or ΔV_h_ = V_h(mSlo1)_ – V_h(STREX-2-e9alt)_ (*magenta*). ∗*p* < 0.05, ∗∗*p* < 0.01, ∗∗∗*p* < 0.001, and ∗∗∗∗*p* < 0.0001. The data are presented as mean ± SE, averaged from n = 4 to 9 patches. V_h_ Details in Table 1. IHC, inner hair cell; STREX, stress-axis regulated exon.

Then, we measured the voltage dependence and Ca^2+^ sensitivity of the mSlo1 (isoform 21 without the STREX exon and exon 9 alt, NP_001240306.1), STREX-1 (isoform 8, NP_034740.2), STREX-2, STREX-1-e9alt (isoform 8 with exon9alt), and STREX-2-e9alt (STREX-2 with exon9alt) by performing inside-out patches in *Xenopus* oocytes. The gate voltages (GVs) of these five constructs were generated using currents recorded in 0 to 100 μM intracellular Ca^2+^ solution in *Xenopus* oocytes and fitted by the Boltzmann equation (Fig. 2, *A–E*). Unexpectedly, the STREX-2 exon confers an approximate 50 mV negative V_h_ shift of the BK channel in 0 [Ca^2+^]_i_, whereas the STREX exon did not produce a shift the V_h_ under the same condition (Fig. 2, *F* and *G*). In all tested Ca^2+^ concentrations (1–100 μM), STREX-2 and STREX-1 exhibited more negative V_h_ shifts than the mslo1, indicating an enhanced Ca^2+^ sensitivity of these two constructs (Fig. 2, *F* and *G*). In contrast, the exon9alt conferred a less negative GV shift to both STREX-1 and STREX-2 in higher [Ca^2+^]_i_ than to the mSlo1, indicating the channel activation of STREX-1-e9alt or STREX-2-e9alt is less sensitive to Ca^2+^ (Fig. 2, *E*, *F* and *H*, Table 1). Between the STREX-1-e9alt and STREX-2-e9alt, the STREX-2-e9alt was less sensitive to intracellular Ca^2+^. Interestingly, both STREX-1-e9alt and STREX-2-e9alt produced a 50-mV leftward V_h_ shift in 0 [Ca^2+^]_i_ (Fig. 2*F* and Table 1).

**Table 1:** The details of the data shown in the experiment

Figure No	The result from statistical analysis (mean ± SEM, n)					
	V_1/2_ (0 Ca)	V_1/2_ (1 Ca)	V_1/2_ (4 Ca)	V_1/2_ (10 Ca)	V_1/2_ (30 Ca)	V_1/2_ (100 Ca)

Fig. 2, *A* and *F*, mSlo1	188.9 ± 2.54, n = 5	144.6 ± 3.69, n = 5	90.9 ± 5.20, n = 5	45.6 ± 3.23, n = 5	27.2 ± 5.27, n = 5	−2.7 ± 3.66, n = 5
Fig. 2, *B* and *F*, STREX-1	189.4 ± 6.01, n = 12	123.4 ± 4.83, n = 12	72.4 ± 4.38, n = 12	−20.2 ± 4.59, n = 12	−37.8 ± 7.06, n = 12	−67.4 ± 5.04, n = 12
Fig. 2, *C* and *F*, STREX-2	142.5 ± 4.25, n = 20	111.8 ± 3.82, n = 20	65.0 ± 3.04, n = 20	−5.8 ± 2.98, n = 20	−31.4 ± 3.38, n = 20	−62.4 ± 3.56, n = 20
Fig. 2, *D* and *F*, STREX-1-e9alt	146.5 ± 10.77, n = 11	126.5 ± 6.01, n = 11	100.8 ± 5.26, n = 11	49.8 ± 4.63, n = 11	35.2 ± 6.20, n = 11	17.2 ± 6.25, n = 11
Fig. 2, *E* and *F*, STREX-2-e9alt	145.9 ± 5.06, n = 9	131.2 ± 4.71, n = 9	105.0 ± 7.46, n = 9	74.3 ± 5.18, n = 9	51.5 ± 5.09, n = 9	41.9 ± 4.29, n = 9

	ΔV_1/2_ (0 Ca)	ΔV_1/2_ (1 Ca)	ΔV_1/2_ (4Ca)	ΔV_1/2_ (10 Ca)	ΔV_1/2_ (30 Ca)	ΔV_1/2_ (100 Ca)

Fig. 2*G*, mSlo1/STREX-1	−0.6 ± 1.98, n = 5	19.5 ± 2.67, n = 9	18. 8 ± 4.06, n = 7	66.6 ± 2.02, n = 4	65.5 ± 6.50, n = 4	64.9 ± 1.52, n = 4
mSlo1/STREX-2	46.4 ± 1.64, n = 6	33.7 ± 2.22, n = 9	25.8 ± 3.63, n = 7	51.8 ± 2.44, n = 4	58.8 ± 3.47, n = 4	59.7 ± 2.94, n = 4
Fig. 2*H*, mSlo1/STREX-1-e9alt	43.8 ± 3.05, n = 6	17.6 ± 2.42, n = 9	−9.4 ± 3.08, n = 8	−5.1 ± 2.02, n = 4	−9.0 ± 1.80, n = 4	−21.3 ± 1.79, n = 4
mSlo1/STREX-2-e9alt	44.0 ± 2.52, n = 7	13.3 ± 1.93, n = 9	−14.8 ± 4.17, n = 9	−31.5 ± 3.74, n = 5	−25.1 ± 4.37, n = 5	−45.5 ± 2.69, n = 5

	V_1/2_ (0 Ca, 0 mmHg)		V_1/2_ (0 Ca, 40 mmHg)		V_1/2_ (0 Ca, 60 mmHg)	

Fig. 3, *D* and *E*, STREX-2	142.5 ± 4.25, n = 4		112.4 ± 4.38, n = 4		92.4 ± 4.79, n = 4	
Fig. 3, *I* and *J*, STREX-2-e9alt	145.9 ± 5.06, n = 4		133.3 ± 9.52, n = 4		112.7 ± 5.18, n = 4	

	V_1/2_ (10 Ca, 0 mmHg)		V_1/2_ (10 Ca, 40 mmHg)		V_1/2_ (10 Ca, 60 mmHg)	

Fig. 3, *D* and *E*, STREX-2	−5.8 ± 2.98, n = 4		−14.9 ± 4.21, n = 4		−34.3 ± 2.57, n = 4	
Fig. 3, *I* and *J*, STREX-2-e9alt	74.3 ± 5.18, n = 4		50.7 ± 8.78, n = 4		38.0 ± 4.66, n = 4	

	V_1/2_ (100 Ca, 0 mmHg)		V_1/2_ (100 Ca, 40 mmHg)		V_1/2_ (100 Ca, 60 mmHg)	

Fig. 3, *D* and *E*, STREX-2	−61.6 ± 3.62, n = 4		−70.8 ± 3.17, n = 4		−81.5 ± 2.38, n = 4	
Fig. 3, *I* and *J*, STREX-2-e9alt	41.9 ± 4.29, n = 4		37.3 ± 8.46, n = 4		9.0 ± 7.13, n = 4	

	ΔV_1/2_ (0 Ca)		ΔV_1/2_ (10 Ca)		ΔV_1/2_ (100 Ca)	
	40 mmHg	60 mmHg	40 mmHg	60 mmHg	40 mmHg	60 mmHg

Fig. 3*K*, STREX-2	30.0 ± 4.46, n = 4	50.0 ± 3.51, n = 4	9.4 ± 4.27, n = 4	28.7 ± 1.59, n = 4	10.1 ± 1.21, n = 4	9.2 ± 1.34, n = 4
Fig. 3*L*, STREX-2-e9alt	12.4 ± 5.41, n = 4	33.0 ± 2.06, n = 4	24.2 ± 4.18, n = 4	36.9 ± 4.83, n = 4	4.9 ± 4.24, n = 4	33.2 ± 5.46, n = 4

	V_1/2_ (0 Ca)	V_1/2_ (1 Ca)	V_1/2_ (4 Ca)	V_1/2_ (10 Ca)	V_1/2_ (30 Ca)	V_1/2_ (100 Ca)

Fig. 4*E*, STREX-1	190.5 ± 7.36, n = 12	124.9 ± 5.62, n = 12	73.5 ± 4.65, n = 12	−21.5 ± 3.11, n = 12	−34.0 ± 6.46, n = 12	−67.4 ± 5.04, n = 12
STREX-1 + LRRC52	91.3 ± 4.39, n = 12	60.6 ± 7.68, n = 12	27.5 ± 6.26, n = 10	−53.1 ± 6.17, n = 11	−106.2 ± 6.66, n = 9	−140.2 ± 6.56, n = 9
Fig. 4*F*, STREX-2	143.6 ± 2.00, n = 20	110.8 ± 2.44, n = 20	64.4 ± 1.69, n = 20	−5.0 ± 1.93, n = 20	−31.6 ± 1.54, n = 20	−62.9 ± 1.46, n = 20
STREX-2+LRRC52	57.3 ± 5.69, n = 7	23.9 ± 5.88, n = 7	−16.6 ± 6.80, n = 7	−64.9 ± 5.63, n = 7	−93.3 ± 7.36, n = 7	−127.0 ± 4.91, n = 7
Fig. 4*G*, STREX-1-e9alt	148.3 ± 8.51, n = 11	125.7 ± 6.34, n = 11	101.1 ± 6.46, n = 11	51.3 ± 2.49, n = 11	38.3 ± 3.11, n = 11	18.6 ± 5.19, n = 11
STREX-1-e9alt+LRRC52	76.5 ± 5.12, n = 5	79.4 ± 3.30, n = 5	61.4 ± 2.61, n = 5	7.03 ± 6.00, n = 5	−35.7 ± 12.74, n = 5	−100.0 ± 16.37, n = 5
Fig. 4*H*, STREX-2-e9alt	145.9 ± 5.06, n = 9	131.2 ± 4.71, n = 9	105.0 ± 7.46, n = 9	74.3 ± 5.18, n = 9	51.5 ± 5.09, n = 9	41.9 ± 4.29, n = 9
STREX-2-e9alt+LRRC52	62.8 ± 1.28, n = 11	59.0 ± 1.68, n = 11	39.8 ± 2.16, n = 15	2.7 ± 1.75, n = 15	−16.4 ± 4.45, n = 15	−22.3 ± 3.15, n = 13

	ΔV_1/2_ (0 Ca)	ΔV_1/2_ (1 Ca)	ΔV_1/2_ (4Ca)	ΔV_1/2_ (10 Ca)	ΔV_1/2_ (30 Ca)	ΔV_1/2_ (100 Ca)

Fig. 4*I*	99.2 ± 3.34, n = 9	64.4 ± 3.77, n = 9	46.0 ± 2.68, n = 10	31.5 ± 3.23, n = 10	72.2 ± 3.56, n = 12	72.8 ± 2.48, n = 12
Fig. 4*J*	72.4 ± 2.81, n = 5	94.1 ± 3.31, n = 8	79.3 ± 5.61, n = 9	54.0 ± 3.09, n = 7	61.6 ± 5.10, n = 8	60.2 ± 3.01, n = 7
Fig. 4*K*	71.7 ± 3.66, n = 5	46.3 ± 3.74, n = 5	39.7 ± 3.85, n = 5	44.3 ± 3.95, n = 5	74.1 ± 9.79, n = 5	118.6 ± 11.40, n = 5
Fig. 4*L*	83.2 ± 3.93, n = 5	72.2 ± 2.72, n = 5	65.1 ± 6.00, n = 5	71.9 ± 4.09, n = 5	68.5 ± 1.94, n = 5	64.1 ± 2.51, n = 5

	V_1/2_ (0 Ca, 0 mmHg)		V_1/2_ (0 Ca, 40 mmHg)		V_1/2_ (0 Ca, 60 mmHg)	

Fig. 5, *D* and *E*, STREX-2+LRRC52	69.5 ± 3.88, n = 4		41.0 ± 2.47, n = 4		34.5 ± 4.79, n = 4	
Fig. 5, *I* and *J*, STREX-2-e9alt+LRRC52	61.0 ± 1.51, n = 4		55.3 ± 1.94, n = 4		26.7 ± 7.83, n = 4	

	V_1/2_ (10 Ca, 0 mmHg)		V_1/2_ (10 Ca, 40 mmHg)		V_1/2_ (10 Ca, 60 mmHg)	

Fig. 5, *D* and *E*, STREX-2+LRRC52	−59.5 ± 4.97, n = 4		−68.0 ± 5.02, n = 4		−86.7 ± 3.01, n = 4	
Fig. 5, *I* and *J*, STREX-2-e9alt + LRRC52	2.7 ± 1.75, n = 4		−8.7 ± 2.56, n = 4		−20.0 ± 3.69, n = 4	

	V_1/2_ (100 Ca, 0 mmHg)		V_1/2_ (100 Ca, 40 mmHg)		V_1/2_ (100 Ca, 60 mmHg)	

Fig. 5, *D* and *E*, STREX-2+LRRC52	−121.5 ± 3.85, n = 4		−132.0 ± 6.20, n = 4		−142.8 ± 4.61, n = 4	
Fig. 5, *I* and *J*, STREX-2-e9alt+LRRC52	−22.3 ± 3.15, n = 4		−32.0 ± 4.28, n = 4		−52.6 ± 4.74, n = 4	

	ΔV_1/2_ (0 Ca)		ΔV_1/2_ (10 Ca)		ΔV_1/2_ (100 Ca)	
	40 mmHg	60 mmHg	40 mmHg	60 mmHg	40 mmHg	60 mmHg

Fig. 5*K*	26.0 ± 2.33, n = 4	34.3 ± 1.88, n = 4	11.9 ± 3.08, n = 4	28.1 ± 4.57, n = 4	16.1 ± 5.73, n = 4	24.5 ± 3.23, n = 4
Fig. 5*L*	4.3 ± 1.06, n = 4	33.7 ± 6.01, n = 4	11.5 ± 1.59, n = 4	22.5 ± 2.25, n = 4	7.9 ± 3.77, n = 4	29.1 ± 2.51, n = 4

Fig. 6*B*	WT			KCNMA1 CKO		
	0.94 ± 0.04, n = 6			0.30 ± 0.06, n = 6		

Fig. 7*B*		Click	4 kHz	8 kHz	12 kHz	16 kHz

	WT	41.0 ± 2.08, n = 10	41.5 ± 1.67, n = 10	34.5 ± 0.90, n = 10	29.5 ± 1.17, n = 10	24.0 ± 1.00, n = 10
	IHC^cre/+^	36.7 ± 2.36, n = 9	45.0 ± 2.64, n = 9	34.4 ± 1.30, n = 9	30.0 ± 1.18, n = 9	25.6 ± 1.30, n = 9
	KCNMA1^flox/flox^	38.1 ± 1.88, n = 8	39.4 ± 1.13, n = 8	35.0 ± 1.34, n = 8	29.4 ± 1.75, n = 8	24.4 ± 1.13, n = 8
	IHC^cre/+^/KCNMA1^flox/flox^	73.0 ± 1.86, n = 10	89.5 ± 1.17, n = 10	68.5 ± 1.07, n = 10	60.0 ± 1.49, n = 10	53.5 ± 1.50, n = 10

	V_1/2_ (0 Ca, 0 mmHg)		V_1/2_ (0 Ca, 40 mmHg)		V_1/2_ (0 Ca, 60 mmHg)	

Sup Fig. 1D, 1E, STREX-1	189.4 ± 6.01, n = 4		130.6 ± 6.45, n = 4		120.5 ± 5.41, n = 4	
Sup Fig. 1I, 1J, STREX-1-e9alt	133.5 ± 8.26, n = 4		84.7 ± 8.67, n = 4		80.7 ± 8.77, n = 4	

	V_1/2_ (10 Ca, 0 mmHg)		V_1/2_ (10 Ca, 40 mmHg)		V_1/2_ (10 Ca, 60 mmHg)	

Sup Fig. 1D, 1E, STREX-1	−20.2 ± 4.59, n = 9		−51.1 ± 6.23, n = 9		−62.9 ± 5.42, n = 9	
Sup Fig. 1I, 1J, STREX-1-e9alt	49.8 ± 4.63, n = 4		13.9 ± 3.79, n = 4		2.8 ± 5.35, n = 4	

	V_1/2_ (100 Ca, 0 mmHg)		V_1/2_ (100 Ca, 40 mmHg)		V_1/2_ (100 Ca, 60 mmHg)	

Sup Fig. 1D, 1E, STREX-1	−67.4 ± 5.04, n = 7		−96.9 ± 6.39, n = 7		−111.6 ± 9.55, n = 7	
Sup Fig. 1I, 1J, STREX-1-e9alt	17.2 ± 6.25, n = 4		−27.3 ± 10.18, n = 4		−38.0 ± 5.34, n = 4	

	ΔV_1/2_ (0 Ca)		ΔV_1/2_ (10 Ca)		ΔV_1/2_ (100 Ca)	
	40 mmHg	60 mmHg	40 mmHg	60 mmHg	40 mmHg	60 mmHg

Sup Fig. 1K	59.3 ± 4.41, n = 4	69.5 ± 3.42, n = 4	30.0 ± 4.12, n = 9	41.8 ± 3.34, n = 9	29.0 ± 3.13, n = 7	43.7 ± 5.68, n = 7
Sup Fig. 1L	46.7 ± 11.66, n = 4	52.6 ± 7.12, n = 4	38.9 ± 1.81, n = 4	47.0 ± 3.11, n = 4	45.9 ± 5.53, n = 4	56.6 ± 0.86, n = 4

	V_1/2_ (0 Ca, 0 mmHg)		V_1/2_ (0 Ca, 40 mmHg)		V_1/2_ (0 Ca, 60 mmHg)	

Sup Fig. 2D, 2E, STREX-1+LRRC52	91.3 ± 4.39, n = 5		70.6 ± 5.29, n = 5		56.0 ± 3.29, n = 5	
Sup Fig. 2I, 2J, STREX-1-e9alt + LRRC52	76.5 ± 5.12, n = 5		56.5 ± 6.00, n = 5		56.0 ± 5.97, n = 5	

	V_1/2_ (10 Ca, 0 mmHg)		V_1/2_ (10 Ca, 40 mmHg)		V_1/2_ (10 Ca, 60 mmHg)	

Sup Fig. 2D, 2E, STREX-1+LRRC52	−53.1 ± 6.17, n = 5		−65.1 ± 6.39, n = 5		−66.7 ± 2.08, n = 5	
Sup Fig. 2I, 2J, STREX-1-e9alt+LRRC52	7.03 ± 6.00, n = 5		−19.2 ± 5.29, n = 5		−31.3 ± 3.73, n = 5	

	V_1/2_ (100 Ca, 0 mmHg)		V_1/2_ (100 Ca, 40 mmHg)		V_1/2_ (100 Ca, 60 mmHg)	

Sup Fig. 2D, 2E, STREX-1+LRRXC52	−140.2 ± 6.56, n = 5		−142.3 ± 4.21, n = 5		−153.9 ± 3.13, n = 5	
Sup Fig. 2I, 2J, STREX-1-e9alt+LRRC52	−100.0 ± 14.64, n = 5		−133.0 ± 8.87, n = 5		−154.5 ± 5.37, n = 5	

	ΔV_1/2_ (0 Ca)		ΔV_1/2_ (10 Ca)		ΔV_1/2_ (100 Ca)	
	40 mmHg	60 mmHg	40 mmHg	60 mmHg	40 mmHg	60 mmHg

Sup Fig. 2K	22.3 ± 1.59, n = 5	36.0 ± 2.08, n = 5	11.1 ± 0.80, n = 5	14.4 ± 2.33, n = 5	2.1 ± 2.21, n = 5	13.6 ± 7.61, n = 5
Sup Fig. 2L	18.6 ± 4.58, n = 5	19.0 ± 4.49, n = 5	26.1 ± 2.43, n = 5	38.4 ± 3.08, n = 5	31.6 ± 10.92, n = 5	53.8 ± 13.29, n = 5

IHC, inner hair cell; STREX, stress-axis regulated exon.

**Figure 3:**
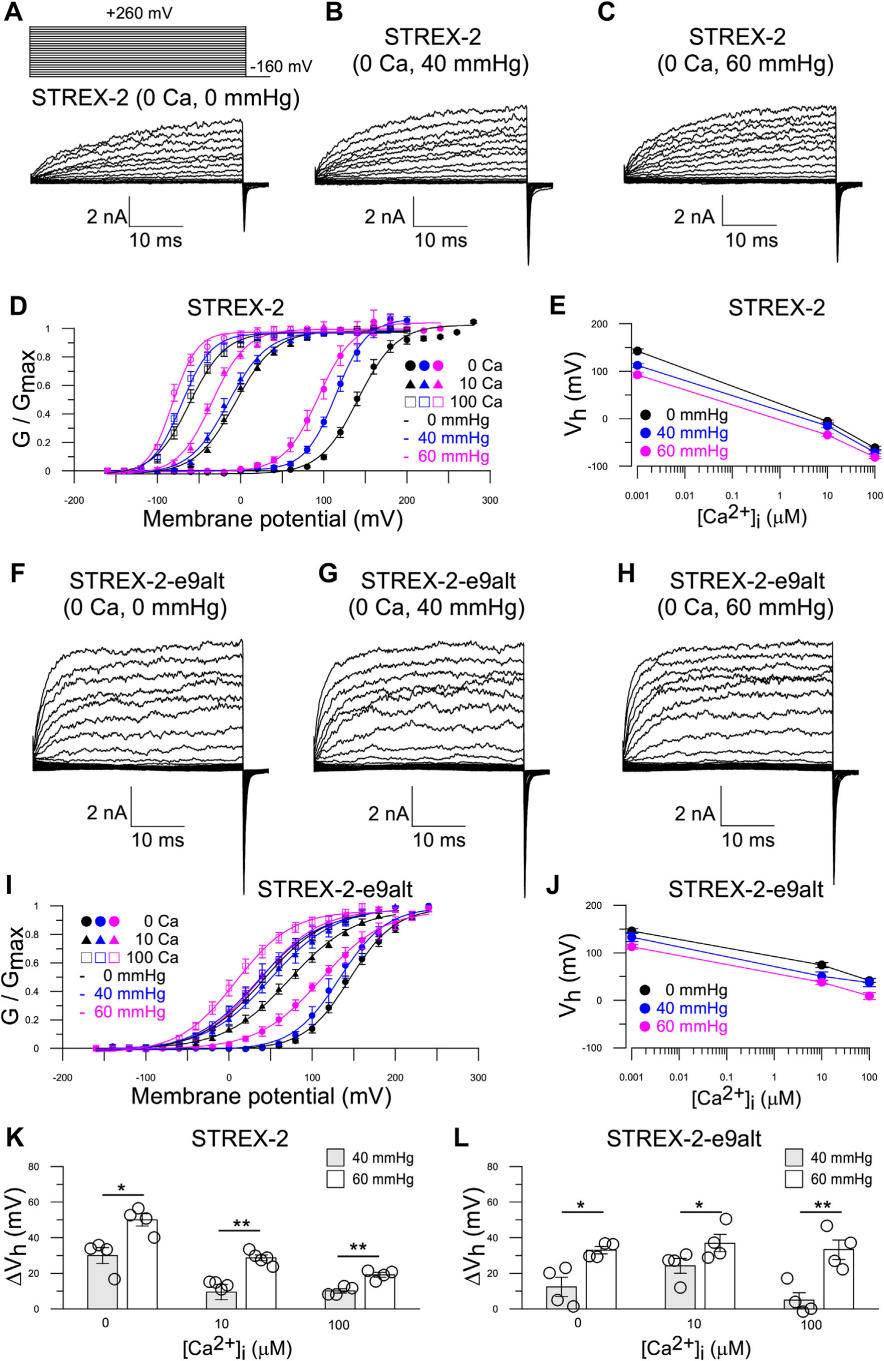
**Effects of stretch on STREX-2 and STREX-2-e9alt variants activity.***A–C,* representative current traces of STREX-2 in 0 mM Ca^2+^ were elicited by stretch at 0 mmHg (*A*), 40 mmHg (*B*), and 60 mmHg (*C*), respectively. *D* and *E,* mean GV relationship (*D*) and V_h_ (*E*) of STREX-2 variant with stretch at 0 mmHg (*black*), 40 mmHg (*blue*), and 60 mmHg (*pink*) in 0 μM (*solid circle*), 10 μM (*solid triangle*), and 100 μM [Ca^2+^]_i_ (*empty square*), respectively. *F–H,* representative current traces of STREX-2-e9alt in 0 mM Ca^2+^ were elicited by stretch at 0 mmHg (*F*), 40 mmHg (*G*), and 60 mmHg (*H*), respectively. *I* and *J,* mean GV relationship (*I*) and V_h_ (*J*) of STREX-2-e9alt channel with stretch at 0 mmHg, 40 mmHg, and 60 mmHg in 0 μM, 10 μM, and 100 μM [Ca^2+^]_i_, respectively. *K,* ΔV_h_ between 0 mmHg and 40 mmHg/60 mmHg of STREX-2 variant at 0 μM, 10 μM, and 100 μM [Ca^2+^]_i_, respectively. At each [Ca^2+^]_i_, ΔV_h_ = V_h (STREX-2, at 0 mmHg)_ – V_h (STREX-2, at 40 mmHg)_ or ΔV_h_ = V_h (STREX-2, at 0 mmHg)_ – V_h (STREX-2, at 60 mmHg)_. ∗*p* < 0.05 and ∗∗*p* < 0.01. The data are presented as mean ± SE, n = 4 patches. *L,* the ΔV_h_ between 0 mmHg and 40 mmHg/60 mmHg of STREX-2-e9alt variant at 0 μM, 10 μM, and 100 μM [Ca^2+^]_i_, respectively. At each [Ca^2+^]_i_, ΔV_h_ = V_h (STREX-2-e9alt, at 0 mmHg)_ – V_h (STREX-2-e9alt, at 40 mmHg)_ or ΔV_h_ = V_h (STREX-2-e9alt, at 0 mmHg)_ – V_h (STREX-2-e9alt, at 60 mmHg)_. ∗*p* < 0.05 and ∗∗*p* < 0.01. The data are presented as mean ± SE, n = 4 patches. Details in Table 1. IHC, inner hair cell; STREX, stress-axis regulated exon.

Since cochlear hair cells transfer mechanical force generated by sound waves into electrical signals, we further tested whether mechanical force influences the activation of STREX-1/STREX-1-e9alt and STREX-2/STREX-2-e9alt. Both STREX and STREX-e9alt isoforms are sensitive to mechanical force. A stretch of 60 mmHg can produce an approximate 70 mV leftward shift in the STREX-1 GV curve in 0 [Ca^2+^]_i_ (Fig. S1, *A–E* and *K*, Table 1). However, intracellular Ca^2+^ can partially offset the activation effect of the mechanical force. In 100 μM [Ca^2+^]_i,_ a 60 mmHg stretch only induces a leftward shift of approximately 44 mV in the STREX-1 GVs (Fig. S1, *D*, *E* and *K*, Table 1). Furthermore, the 60 mmHg stretch produces an additional 55 mV V_h_ shift in STREX-1-e9alt in 0 [Ca^2+^]_i_, indicating an additive activation effect of the stretch and e9alt (Fig. S1, *I–K*, Table 1). A similar activation effect of the stretch is also observed in STREX-2, and the 60 mmHg stretch force generates an approximate 50 mV V_h_ shift in 0 [Ca^2+^]_i_, but only a 20 mV shift in 100 μM [Ca^2+^]_i_ (Fig. 3, *A–E* and *K*, Table 1). In contrast, the STREX-2-e9alt demonstrates less stretch activity, with a 60 mmHg stretch resulting in only a 30 mV leftward shift in the GV curve across all [Ca^2+^]_i_ concentrations (Fig. 3, *F–J* and *L*). Additionally, since the LRRC52 subunit has been shown to be expressed in cochlear IHCs, it is important to determine whether the stretch-induced GV shifts are maintained when the α subunit and LRRC52 are coexpressed under physiological conditions.

**Figure 4:**
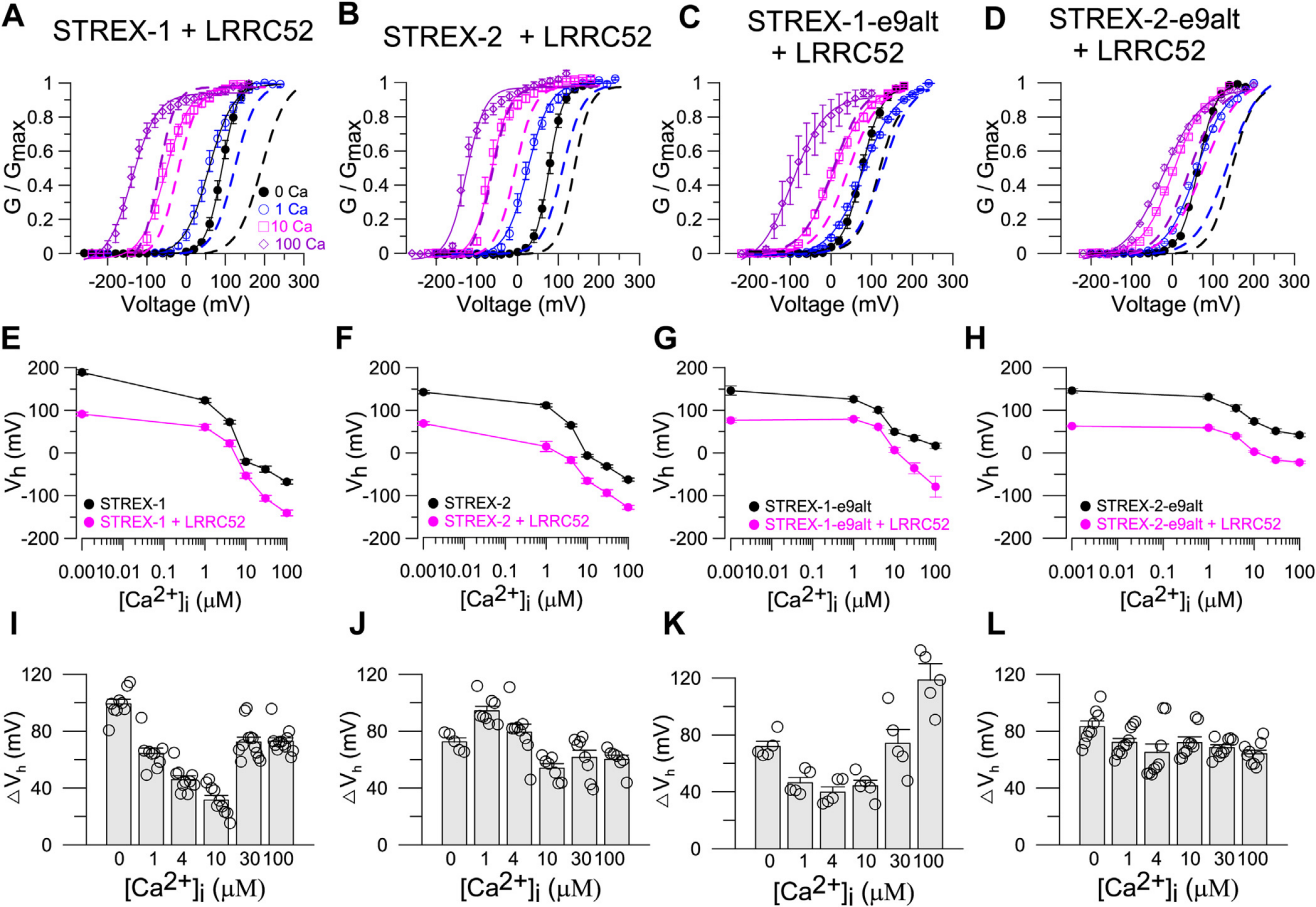
**Effects of coexpression with LRRC52 subunit on the activity of STREX-1, STREX-2, STREX-1-e9alt, and STREX-2-e9alt.***A–E,* normalized GV curves for STREX-1 + LRRC52 (*A*), STREX-2+LRRC52 (*B*), STREX-1-e9alt+LRRC52 (*C*), and STREX-2-e9alt + LRRC52 (*D*) channels in 0∼100 μM [Ca^2+^]_i_ were presented with *dashed lines*. Normalized GV curves of STREX-1 (*A*), STREX-2 (*B*), STREX-1-e9alt (*C*), and STREX-2-e9alt (*D*) channels in 0∼100 μM [Ca^2+^]_i_ were presented with *solid lines*. All GV relationships are fitted with the Boltzmann relation. *E,* the V_h_-[Ca^2+^]_i_ relationship of STREX-1 and STREX-1+LRRC52. V_h_ is the voltage where the GV relation is half-maximum. *F,* the V_h_-[Ca^2+^]_i_ relationship of STREX-2 and STREX-2+LRRC52 channels. *G,* the V_h_-[Ca^2+^]_i_ relationship of STREX-1-e9alt and STREX-1-e9alt+LRRC52 channels. *H,* the V_h_-[Ca^2+^]_i_ relationship of STREX-2-e9alt and STREX-2-e9alt+LRRC52 channels. *I,* the ΔV_h_ between STREX-1 and STREX-1+LRRC52 in 0∼100 μM [Ca^2+^]_i_. At each [Ca^2+^]_i_, ΔV_h_ = V_h (STREX)_ – V_h (STREX + LRRC52)_. The data are presented as mean ± SE, n = 9 to 12 patches. *J,* the ΔV_h_ between STREX-2 and STREX-2+LRRC52 in 0∼100 μM [Ca^2+^]_i_. At each [Ca^2+^]_i_, ΔV_h_ = V_h (STREX-2)_ – V_h (STREX-2 + LRRC52)_. The data are presented as mean ± SE, n = 5 to 9 patches. *K,* ΔV_h_ between STREX-1-e9alt and STREX-1-e9alt+LRRC52 in 0 ∼ 100 μM [Ca^2+^]_i_. At each [Ca^2+^]_i_, ΔV_h_ = V_h (STREX-e9alt)_ – V_h (STREX-e9alt + LRRC52)_. The data are presented as mean ± SE, n = 5 patches. *L,* ΔV_h_ between STREX-2-e9alt and STREX-2-e9alt+LRRC52 in 0∼100 μM [Ca^2+^]_i_. At each [Ca^2+^]_i_, ΔV_h_ = V_h (STREX-2-e9alt)_ – V_h (STREX-2-e9alt + LRRC52)_. The data are presented as mean ± SE, n = 5 patches. Details in Table 1. IHC, inner hair cell; STREX, stress-axis regulated exon.

We thus further examined the effect of a stretch on the GV shifts when these isoforms were coexpressed with the LRRC52 subunits. The LRRC52 produced leftward shifts in the GVs of all isoforms (Fig. 4, *A–H*, Table 1), although some differences were observed. The leftward V_h_ shift of STREX-1+LRRC52 was less pronounced than that of the STREX-2+LRRC52 in low [Ca^2+^]_i_ (1–10 μM), (Fig. 4, *A*, *B*, *E*, *F*, *I* and *J*). A similar reduction in shift was observed when comparing the V_h_ shifts of STREX-1-e9alt+LRRC52 to those of STREX-2-e9alt+LRRC52. (Fig. 4, *C*, *D*, *G*, *H*, *K* and *L*).

**Figure 5:**
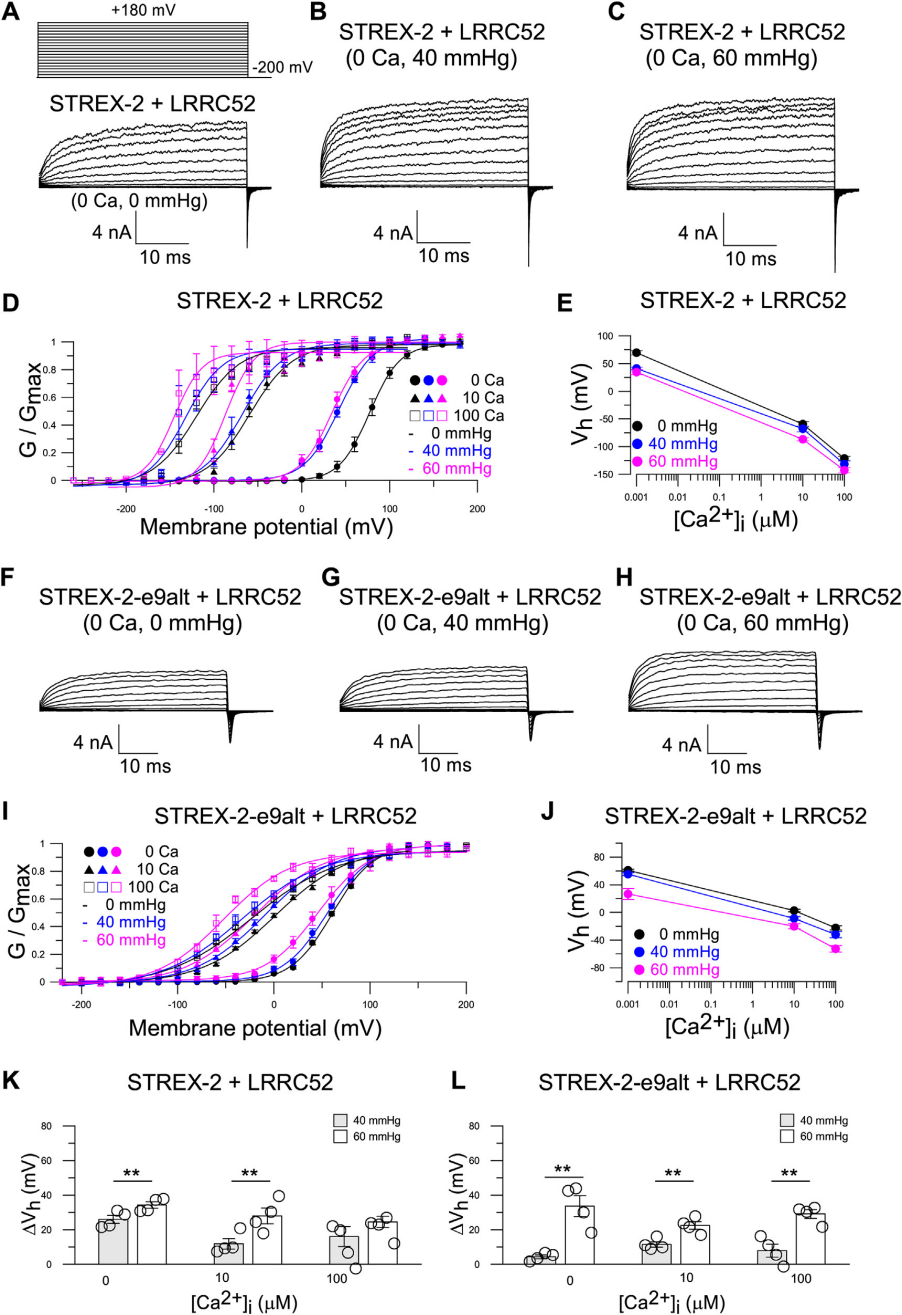
**The mechanical force causes further GV shift of the STREX-2 and STREX-2-e9alt variants with coexpression of the LRRC52 subunit.***A–C,* typical currents were activated with the indicated voltage protocol from −200 mV to +180 mV. Representative current traces of STREX-2+LRRC52 in 0 μM Ca^2+^ elicited by stretch at 0 mmHg (*A*), 40 mmHg (*B*), and 60 mmHg (*C*), respectively. *D–E,* mean GV relationship (*D*) and V_h_ (*E*) of STREX-2+LRRC52 channel with a stretch at 0 mmHg (*black*), 40 mmHg (*blue*), and 60 mmHg (*pink*) in 0 μM, 10 μM, and 100 μM [Ca^2+^]_i_, respectively. *F–H,* representative current traces of STREX-2-e9alt+LRRC52 in 0 μM Ca^2+^ elicited by stretch at 0 mmHg (*F*), 40 mmHg (*G*), and 60 mmHg (*H*), respectively. *I* and *J,* mean GV relationship (*I*) and V_h_ (*J*) of STREX-2-e9alt+LRRC52 channel with a stretch at 0 mmHg, 40 mmHg, and 60 mmHg in 0 μM, 10 μM, and 100 μM [Ca^2+^]_i_, respectively. *K,* ΔV_h_ between 0 mmHg and 40 mmHg/60 mmHg of STREX-2+LRRC52 channel in 0 μM, 10 μM, and 100 μM [Ca^2+^]_i_, respectively. At each [Ca^2+^]_i_, ΔV_h_ = V_h (0 mmHg)_ – V_h (40 mmHg)_ or ΔV_h_ = V_h (0 mmHg)_ – V_h (60 mmHg)_. ∗∗*p* < 0.01., n = 4 patches. *L,* ΔV_h_ between 0 mmHg and 40 mmHg/60 mmHg of the STREX-2-e9alt+LRRC52 channel in 0 μM, 10 μM, and 100 μM [Ca^2+^]_i_, respectively. *p* < 0.01. n = 4 patches. Details in Table 1. IHC, inner hair cell; STREX, stress-axis regulated exon.

When coexpressed with LRRC52, the mechanical force still produces additional leftward shifts in the GVs of STREX-1 and STREX-1-e9alt channels toward negative potentials. For the STREX-1+LRRC52 construct, 40 and 60 mmHg stretch produce approximately 22 and 36 mV leftward V_h_ shift in 0 [Ca^2+^]_I_, respectively; however, they only result in approximately 11 and 14 mV V_h_ shift in 10 μM [Ca^2+^]_i_, and 2 and 14 mV shift in 100 [Ca^2+^]_i_, respectively (Fig. S2, *A–E* and *K*, Table 1). These results indicate that increasing intracellular Ca^2+^ concentrations partially offset the V_h_ shifting effect induced by the mechanical force. This offset effect, however, is not observed in the STREX-1-e9alt+LRRC52 construct. For STREX-1-e9alt+LRRC52, the 40 and 60 mmHg stretches cause approximately 20 mV leftward V_h_ shift in 0 [Ca^2+^]_i_, while they induce 30 and 50 mV V_h_ shift in 100 [Ca^2+^]_i,_ respectively (Fig. S2, *F–H* and *L*, Table 1). Similarly, mechanical force also produces leftward shifts in the GVs of the STREX-2+LRRC52, with a 60 mmHg stretch force induces additional 35 mV leftward V_h_ shift (Fig. 5, *A–E* and *K*, Table 1). Likewise, the mechanical force also further leftward shifts the V_h_ of the STREX-2-e9alt+LRRC52 channel. A 60 mmHg stretch force produces around a 30 mV leftward shift of V_h_ in STREX-2-e9alt+LRRC52 in all tested [Ca^2+^]_i_ concentrations, resulting in a V_h_ of approximately 27 mV in 0 [Ca^2+^]_i_, which is close to the V_h_ measured in IHCs in inside-out patch configuration (Fig. 5, *F–J* and *L*, Table 1).

**Figure 6:**
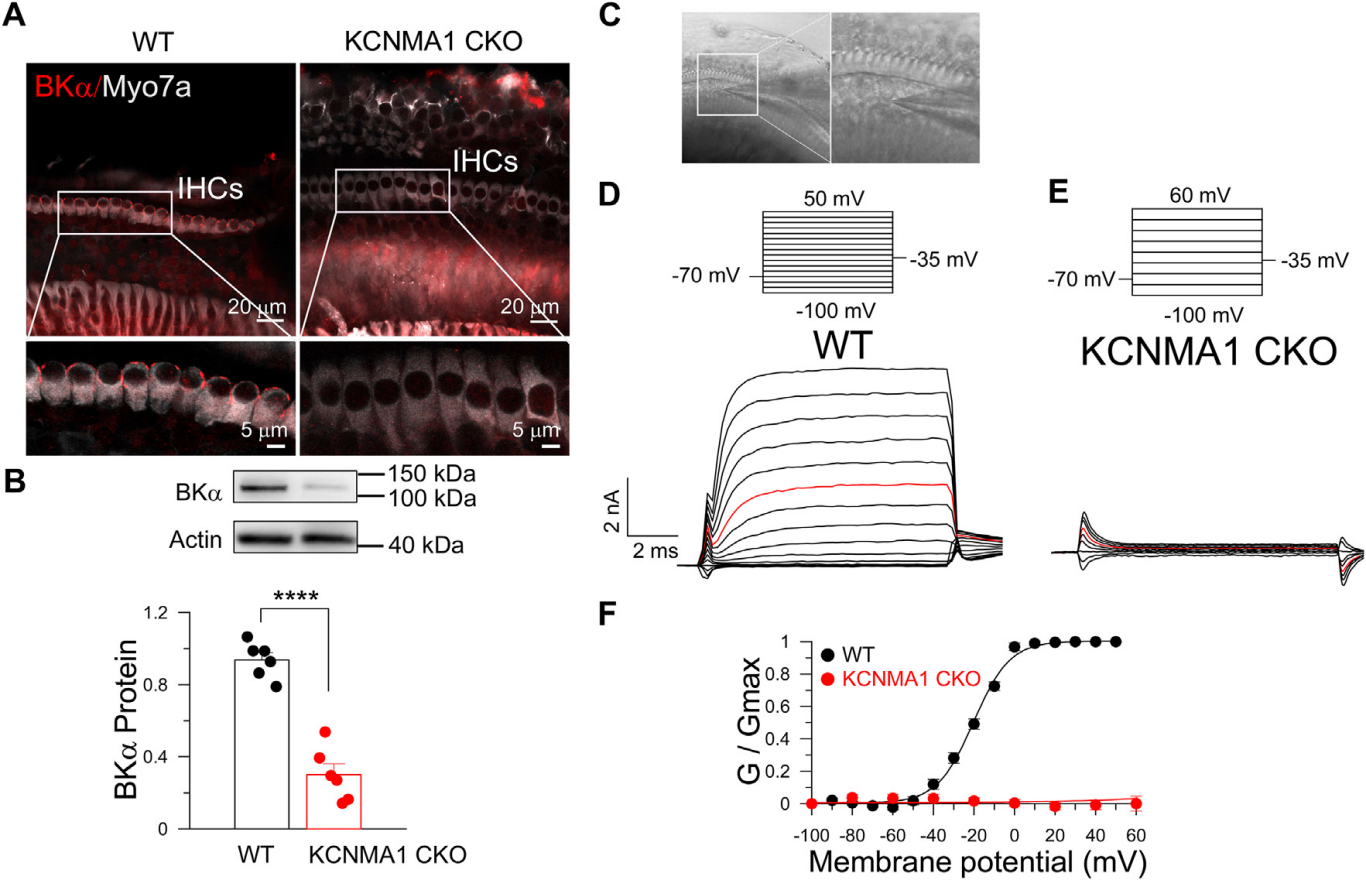
**KCNMA1 CKO mice exhibit loss of BK currents in IHC cells.***A,* confocal images of immunofluorescent staining of KCNMA1 in WT mice and KCNMA1 CKO mice in mice cochlea. *Up:* 20 μm scale bar showing cochlear. *Down:* the image showing the IHC cells. *B,* the KCNMA1 protein expression level in the cochlea of WT mice and KCNMA1 CKO mice. The two-tail unpaired *t* test in (*B*), n = 6, ∗∗∗∗*p* < 0.0001. *C,* the patch formed on the membrane of IHCs in the acutely isolated cochlea explants. *D,* representative traces show the effect of injected voltage (−100 ∼ +50 mV, step +10 mV) induced BK currents in the IHC from the WT mice. *E,* representative traces show no BK currents in the IHC recording with voltage steps (−100 ∼ +60 mV, step +20 mV) from the KCNMA1 CKO mice. *C* and *D, red trace*, step to 0 mV. *F,* normalized conductance-voltage plots obtained from the analysis of steady-state (as in *C*, *D*) with Boltzman fits. For WT, V_h_ = −20.6 ± 1.41 mV, mean values from 7 IHC cells (±SEM) of 4 WT mice (3–8 week old). For KCNMA1 CKO, recorded from 7 IHC cells of 4 KCNMA1 CKO mice (3–8 week old). IHC, inner hair cell; CKO, conditioned KO.

To compare the GVs of the IHC BK channel in 0 [Ca^2+^]_i_ with those of BK isoforms in oocytes, we recorded the original BK channels’ current in IHCs with a whole-cell configuration. Cochleae from mice were isolated, and IHCs were selected and recorded (Fig. 6*A*). To establish a comparison with the BK current recorded in the IHCs of WT mice, the BK channel-deleted IHCs were used. These hair-cell-specific BK channel deleted mice were generated by breeding Atoh1-cre mice with mice carrying the floxed Slo1 gene. The deletion of the BK channel in IHCs was confirmed by Western blot (Fig. 6*B*). The WT BK channel currents were measured from −100 mV to 50 mV, and the GVs were generated using steady-state current and plotted (Fig. 6*C*). The GVs were fitted by the Boltzmann equation, yielding a V_h_ of −20 mV in WT mice. No BK currents could be recorded in the IHCs from Slo1-deleted mice (Fig. 6*D*). To further validate the presence of BK currents, we compared the IHC whole-cell currents before and after perfusion with an extracellular solution containing 200 nM iberiotoxin (IBTX). After perfusion with 200 nM IBTX, most whole-cell currents were inhibited (Fig. S3, *A* and *B*). The GV curves calculated by subtracted BK currents still showed approximately −20 mV V_h_ (Fig. S3, *C* and *D*). These results indicated that the V_h_ of the isoform we cloned in 0 [Ca^2+^]_i_ is still more positive than the V_h_ of the IHC BK channels recorded *via* whole-cell recordings.

**Figure 7:**
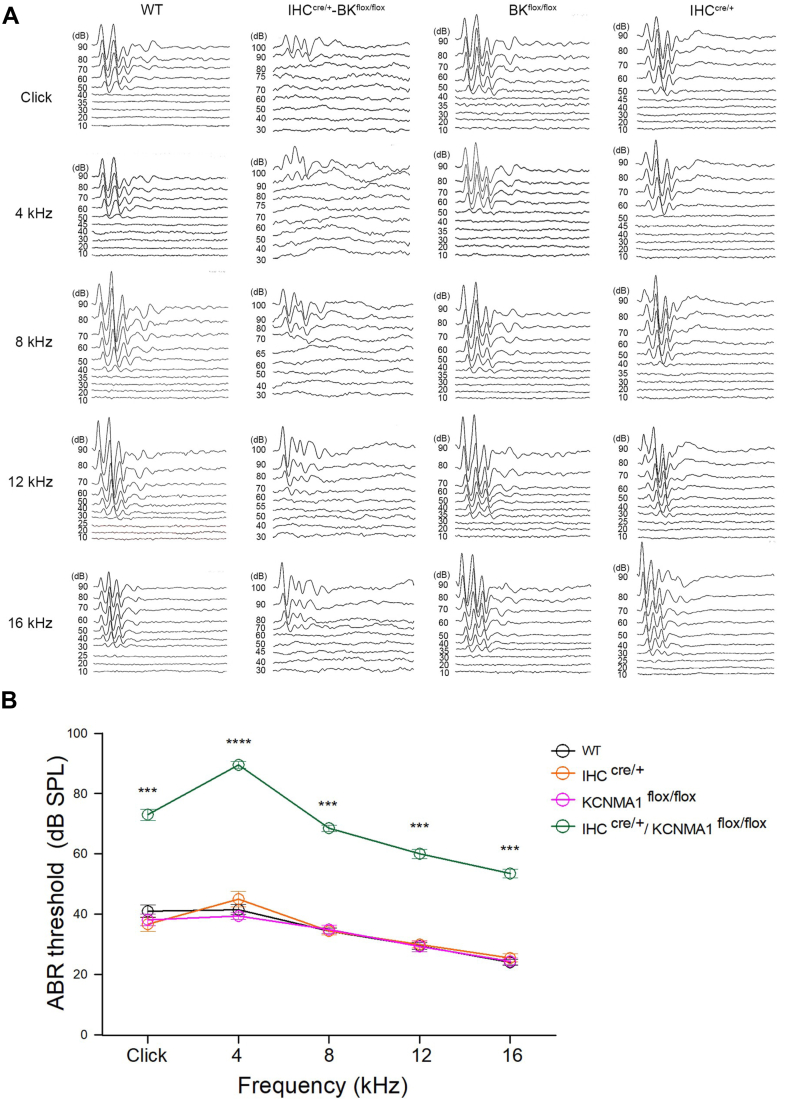
**Abnormal cochlear function in KCNMA1 CKO mice.***A,* representative waveforms of auditory brainstem response (ABR) from WT mice, IHC^cre/+^ mice, KCNMA1^flox/flox^ mice, and IHC^cre/+^/KCNMA1^flox/flox^ (KCNMA1 CKO) mice at different tone frequencies, such as Click, 4, 8, 12, 16 kHz. *B,* average Abr thresholds in WT mice, IHC^cre/+^ mice, KCNMA1^flox/flox^ mice, and IHC^cre/+^/KCNMA1^flox/flox^ (KCNMA1 CKO) mice were recorded at different tone frequencies. For WT (n = 10, 8–12 week old), for IHC^cre/+^ (n = 9, 8–12 week old), KCNMA1 ^flox/flox^ (n = 8, 8–12 week old), for IHC^cre/+^/KCNMA1^flox/flox^ (n = 10). The one-way ANOVA analysis in (*B*), ∗∗∗*p* < 0.001, ∗∗∗∗*p* < 0.0001. Details in Table 1. CKO, conditional KO; IHC, inner hair cell.

Although the BK current is the predominant potassium current in the cochlear IHCs, the role of the BK channel in auditory perception remains a topic of debate. To investigate this, we examined the ABR using IHC-specific BK channel–deleted mice. The results of ABR test revealed a significant increase in the hearing thresholds (from 24–41 dB to 53–73 dB) at click and 4, 8, 12, and 16 kHz tones in the IHC-specific BK channel–deleted mice. The ABR thresholds in these mice were significantly higher than those of WT, LoxP-flanked Slo1, and Atoh1-Cre mice across all tested frequencies (Fig. 7, *A* and *B*). Additionally, there were no significant differences in hearing range between the male and female mice. These findings indicate that the BK channel plays a crucial role in maintaining normal cochlear function.

## Discussion

In this study, we found the additive effect of the exon9alt and STREX alternative splicing of the BK channel, as well as the impact of stretch mechanical force and coexpression of LRRC52 on the leftward shift in GVs of the BK channel in the absence of Ca^2+^. This additive effect can shift the V_h_ (27 mV) of the BK channels expressed in *Xenopus* oocytes closer to the V_h_ (11 mV) of the BK channel recorded in IHCs in the inside-out excised patches (23), but it remains 40 to 60 mV higher than the V_h_ (−20 to 40 mV) of the BK channels recorded in the whole-cell configurations in IHCs (24). In addition, the loss of the BK channel in the IHCs results in a significantly increased threshold for ABR.

Although many splicing isoforms of the BK channels have been detected in the IHCs in previous studies, their translation and biophysical properties have not been fully characterized. One of the isoforms of the BK channel in the IHCs contains a STREX exon, which confers mechanical sensitivity to the BK channel. However, the STREX exon exists in two versions, one is found in the mslo1 isoform 8 (NP_034740.2), named STREX-1 (Fig. 1) (25), while the other, which we refer to as STREX-2, is a variant of mslo1 isoform X5 and includes an extra exon (exon 21) that adds LIYS residues between exon 20 and exon 22 (19, 25).

Interestingly, compared to the mSlo1 isoform 21(NP_001240306.1, referred to as mSlo1) and isoform 8, the STREX-2 exon confers an almost 50 mV leftward GV shift to the BK channel in 0 [Ca^2+^]_i_. This effect is maintained when the α subunit includes exon9alt and is coexpressed with the LRRC52 subunit (Fig. 2*F*, Fig. 4, *F* and *H*). For the Slo1 α subunit containing both the exon9alt and STREX-2 exons, the channel becomes less sensitive to Ca^2+^, yet maintains the leftward shift in 0 [Ca^2+^]_i_, indicating a similar behavior to the BK channels recorded in IHCs.

Furthermore, the V_h_ of STREX-2-e9alt+LRRC52 combination under 60 mmHg stretch in *Xenopus* oocytes is close to the V_h_ of the BK channels recorded in excised IHCs. This suggests that the additive effects of mechanical force, coexpression with LRRC52, and the specific isoform can mimic the biophysical properties of the BK channels in IHCs. However, this raise the question: why is the V_h_ of the BK channel in IHC recorded in excised patches still 40 to 60 mV higher than the V_h_ using whole-cell configuration in the IHCs? One possible explanation is that the BK channels in whole-cell recordings received higher mechanical force than those in excised patches, which can further leftward shift the GV of BK channels in IHCs.

In the physiological condition, the IHCs undertake the responsibility of converting auditory and vestibular stimuli into electrical signals (36). To achieve this conversion, hair cells possess a set of 20 to 300 modified stereocilia, located at the cellular apex and presenting a stereotyped array. Each stereocilium is formed by a bundle of actin filaments and is inserted at the apical end of the hair cell. Stereocilia connect to their adjacent stereocilia through filaments that connect the tip of each stereocilium to the lateral wall of its tallest neighboring stereocilium, termed tip links. During sound stimulation, sound waves provide a mechanical force to deflect hair cells by tensing the tip links. Tip links bind to and deform the membrane to directly gate mechano-transduction channels at their lower ends (37, 38). The effective pressure, calculated as the force in the tip link divided by the height of the cilium, on IHC cilia at the base could reach 3000 Kpa, which is 375-fold higher than the mechanical force (60 mmHg) we exerted on the *Xenopus* membrane (39). Although the tip link does not directly interact with BK channels, the force transferred from the tip link results in hair bundle deflections and causes stereocilia to pivot about the base creating a shearing force on the apical membrane of IHCs (40), which may alter the activity of BK channel through the mechanical sensitivity of STREX exon. The actin-bundling proteins TRIOBP and ESPINS, as critical parts of the stereocilia rootlet, may be involved in this mechanical transduction process by regulating hair bundle compliance (41, 42). Thus, it is possible that the BK channels in IHC cell receive higher mechanical force than the force they experience when they are patched.

When whole-cell recording being performed in the isolated organ of Corti, the pulling force from the patching pipette pulls the cell body from a side without stereocilia, which produces a deforming and displacement effect on the IHCs. Since the microstructures between the hair cells and tip link filament are maintained during seal formation, the mechanical force maybe transferred through the stereocilia to the tectorial membrane because the stereocilia are embedded in the tectorial membrane (43). Thus, the BK channels in IHC cells may be stretched from both sides and experience greater stretch force than the force received by the BK channels in excised patches. Another unexplored possibility is that bundle deflection leads to distortions in the lipid bilayer, such that BK channel gating is correlated with this distortion (40). In excised patches from *Xenopus* oocytes, 60 mmHg represents nearly the highest pressure we can apply to the membrane, as higher pressures often disrupt the patches. However, in whole-cell recordings of IHCs, internal mechanical interactions, such as shearing forces and membrane distortions, may be preserved, allowing BK channels in the IHCs to experience greater pressure than that applied to the membrane of the oocytes, which could further leftward shift their GVs.

**Figure 8:**
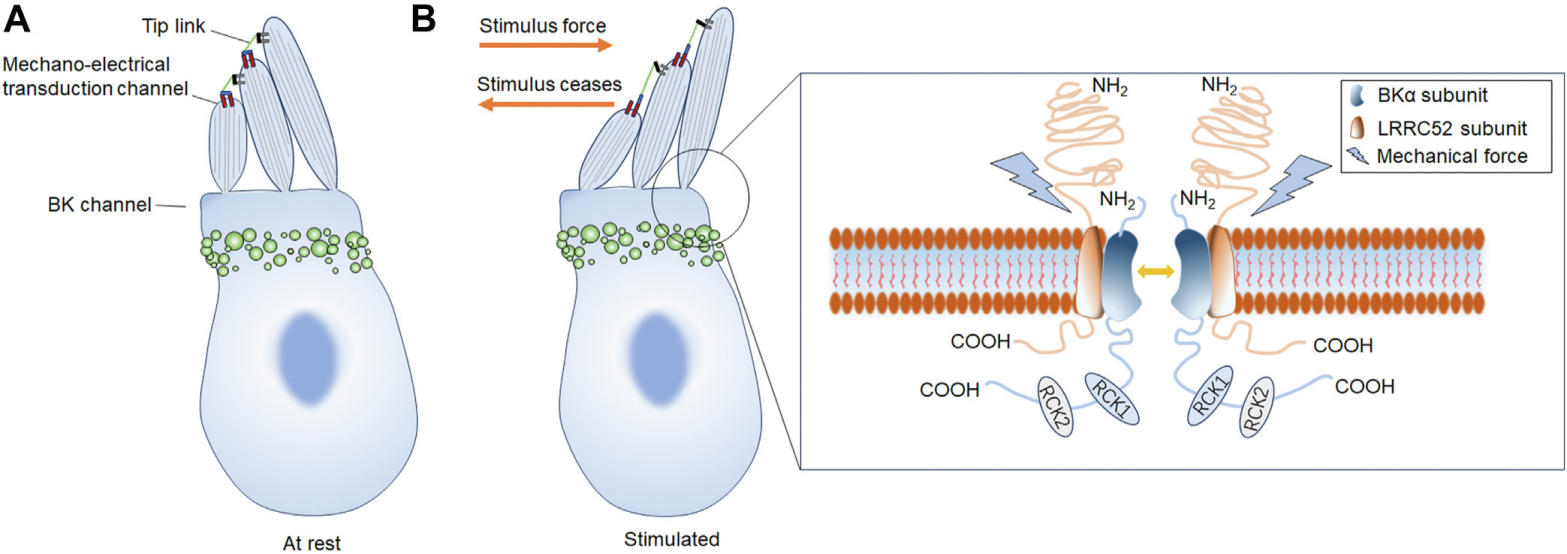
**The scheme illuminates the model of BK channel activation is regulated by mechanical force in IHCs**. *A,* the hair cells are in a rest state. *B,* the hair cells under sound wave stimulation. Inset: mechanical force may pull the channel open through LRRC52/Slo1 and other protein complex. IHC, inner hair cell.

One observation that seems to contradict this explanation is that in LRRC52 KO mice, the GVs of BK channels obtained through whole-cell recording are similar to those recorded in *Xenopus* oocytes (24). However, it is noteworthy that the V_h_ of BK channels in 0 [Ca^2+^]_i_ recorded in IHCs from LRRC52 KO mice is approximately 150 mV, whereas the V_h_ recorded in oocytes is higher than 170 mV. This V_h_ value is consistent with the V_h_ (145 mV) of the STREX-2-e9alt isoform in 0 [Ca^2+^]_i_ that we recorded, suggesting that the BK channels in IHC are likely this isoform. Furthermore, one possibility is that LRRC52 is also required for mechanical force transmission in IHCs (Fig. 8, *A* and *B*), and this transmission is disrupted in LRRC52-deficient mice. One of the LRRC proteins, LRRC10, has been shown to respond to pressure and interact with actin (44). How the mechanical force activates the mechano-sensitive BK channel is still unclear. Previous studies demonstrate that the BK channel can interact with receptor for activated C-kinase 1, which can form an eukaryotic translation initiation factor 6–receptor for activated C-kinase 1–extracellular signal-regulated kinase 1/2–focal adhesion kinase mechanocomplex to regulate mechanical response in endothelial cells (45, 46). However, whether the activation of the BK channel in IHCs is regulated by this mechanism requires further research. The LRRC52 may also be involved in transducing the mechanical force to regulate the GV shifts of the BK channel (Fig. 8*B*).

Another finding in this study is that the conditional deletion of the BK channel in IHCs results in an increased ABR threshold. In a previous study, the authors reported normal ABR thresholds following the deletion of IHC Slo1 using Slo1^−/−^ mice (31). In another study, age-related progressive high-frequency hearing loss was observed in Slo1^−/−^ mice (29). However, all these results were obtained from global Slo1^−/−^ mice, indicating that the findings reflect the consequences of BK deletion in both IHCs and auditory neurons. Therefore, it is possible that the phenotype resulting from BK channel deletion in IHCs was compensated by the absence of BK channels in neurons, as BK currents can limit action potential (AP) broadening, reduce excitatory postsynaptic potentials, and restrict repetitive firing of neurons (1). Thus, the global knockout of BK channels may decrease the acoustic signals from IHCs while enhancing their transmission within the nervous system. This may explain why the hearing loss phenotype in IHC BK-deleted mice is more severe than in global BK-deleted mice (29). However, we do not understand why our results differ from another study that used BK exon 1–deleted mice. Our electrophysiological recordings in IHCs also did not identify the inactivation current recorded after IBTX perfusion (Fig. S3) (31). The remaining exons could theoretically form a truncated Slo1 channel after exon 1 deletion, provided normal splicing is maintained. We noted that there is a methionine amino acid near the N terminal of exon 2, but the authors presented a Western blot confirming that no truncated Slo1 protein was formed (47). In this study, we employed a strategy to remove exons 4 and 5 of Slo1 to disrupt the voltage sensor and pore domain (Fig. S4). Another study that used hair cell-specific BK channel deletion mice found that both peak and steady-state firing rates of auditory nerve fibers were reduced to nearly 50% of control levels (48). However, their hair cell–specific BK deletion mice were generated by breeding prestin-cre mice with floxed Slo1 mice (48). Several studies have indicated that the prestin protein is expressed in outer hair cells (49–51). Since BK channels are typically expressed in IHCs, this strategy may not effectively delete BK channels in IHCs. In our study, we provided all original traces of the ABR recordings and found that the thresholds were increased at all tested frequencies. Additionally, the age of the mice we used ranged from 8 to 12 weeks, but we did not find an age-dependent hearing loss. The Atoh1 gene guides cochlear hair cell development and Atoh1-cre mice have been used for many years to study gene function in IHCs (52–54). Therefore, we believe our results are reliable. Unfortunately, our conditional KO (CKO) mice cannot be utilized to evaluate the role of the STREX exon in hearing loss, which necessitates new mouse models for investigation.

In summary, our results address the role of mechanical force, special isoforms of the Slo1 α subunits, and LRRC52 in the formation of unique biophysical properties of the BK channels in the IHCs and reveal the essential role of the BK channels in the regulation of the auditory perception.

## Experimental procedures

### Animals

All animal protocols and experiments in mice and *Xenopus* were approved by the Institutional Animal Care and Use Committee of Xuzhou Medical University. KCNMA1^fl/fl^ mice were purchased from the GemPharmatech Company. Atoh1-cre mice were gifts from Professor Renjie Chai (School of Life Sciences and Technology, Southeast University). KCNMA1^fl/fl^ and Atoh1-cre mouse lines were backcrossed with the C57BL/6J line for at least six generations. Then, KCNMA1^fl/fl^ mice were crossed with Atoh1-cre mice to generate KCNMA1 CKO mice. The Atoh1-cre and KCNMA1^fl/fl^ were both used as control mice in this study. For routine genotyping, tail DNA from offspring was screened using the following primer sequences: Atoh1-cre (5′- TGCAACGAGTGATGAGGTTC-3′ and 5′- ACGAACCTGGTCGAAATCAG-3′); KCNMA1^flox^ (5′- CACACATGAAGCCCATTAACCTGC-3′ and 5′- AAACCCTATGTCCTCCAGCCCATT-3′).

### The BK channel isoforms identification and sequence confirmation

Total RNA was extracted from the cochleae of C57BL/6 mice using an RNAiso Plus Kit (TAKARA, CAS No. 108-95-2) according to the manufacturer’s protocol. First-strand complementary DNA synthesis was performed using the SuperScript First-Stand Synthesis System for RT-PCR (Invitrogen, 11904018) with an oligo d (T) primer. The exon9alt gene was identified by PCR using the cochleae complementary DNA extracted from C57BL/6 mice as a template. The primers for amplifying the exon9alt segment were F: 5′-TCGGCGGGACTTTTAACAAACATG-3′ and R: 5′-CGAAGGCACTGGAGAGATATTC-3′. The PCR product was sequenced by the Genewiz cooperation. The LRRC52 was a gift from Professor Christopher Lingle (Washington University in Saint Louis).

The channel constructs were modified from the clone of the mouse Slo1 isoform 21 and isoform 8 (NP_034740.2). The STREX-2 was generated by mutagenesis using isoform 8 as a template. The mutagenesis was performed by PCR using Phanta EVO Super-Fidelity DNA polymerase (Vazyme, P503-d1). All the isoforms were cloned into the pXMX vector and sequenced.

### The cRNA synthesis and microinjection

The variants of the BK channels were cloned into the pXMX vector and linearized by mluI endonuclease. The RNA was transcribed *in vitro* using Sp6 polymerase, respectively. 15 to 20 ng RNA was injected into each *Xenopus laevis* oocyte 3 to 7 days before recording. For the mixture experiments, the α subunits and γ subunits were mixed with a 1:2 M ratio.

### ABR measurement

The hearing ability of the mice was measured *via* the ABR). Briefly, 8 to 12 weeks old mice were anesthetized with an intraperitoneal injection of 1.2% pentobarbital sodium (60 mg/kg), and the temperature of the mice was maintained at 37 °C using a heating pad. ABRs were measured with a tone pip stimulus (click, 4 kHz, 8 kHz, 12 kHz, and 16 kHz), using NEURO-Audio/2-channel ABR System (Neurosoft) and Neuro-Audio.NET software. ABRs were recorded with four stainless steel needle electrodes inserted subcutaneously into the position as follows: one was inserted at the top of the skull between the ears of mice, two were inserted at the position below the left and right auricles, and one was inserted at the above dorsal midline of the mouse tail, respectively. For each frequency, the intensity of sound varied from 90 dB to 10 dB, decreasing the sound pressure level first in 10 dB steps and then up and down in 5 dB steps to identify the lowest level at which an ABR pattern could be recognized. ABR measurement was performed in each group of mice using a blinded procedure. The statistical analysis among different groups was performed using one-way ANOVA.

### *Xenopus* oocytes and organ of corti explants preparation

Stage IV *X. laevis* oocytes were prepared according to protocols used in this laboratory (47, 48). All the protocols we used followed Xuzhou Medical University’s Institutional Animal Care and Use Committee guidelines. Adult female *X. laevis* frogs were anesthetized by tricaine and performed on ice. A small cut was made on one side of the abdomen of the frog to remove several pieces of ovarian lobes under aseptic conditions. The lobes were washed and placed in a sterile OR2 solution (in mM, 85 NaCl, 5 KCl, 5 Hepes-NaOH, and 1 MgCl_2_, pH 7.0) at room temperature for 45 min and washed in ND96 solution (in mM, 96 NaCl, 2 KCl, 1.8 CaCl_2_, 1 MgCl_2_, 2.5 Na pyruvate, 5 HEPES, pH 7.5) at room temperature for another 1 h on a rotator (30 rpm).

Organ of Corti explants were acutely isolated from 3 to 8 week male and female mice in the following cutting solution (in mM): 144 NaCl, 5.8 KCl, 0.9 MgCl_2_, 1.3 CaCl_2_, 0.7 NaH_2_PO_4_, 10 Hepes, 5.6 D-Glucose, pH adjusted to 7.4 with NaOH, osmolarity at 290 to 310 mOsm. Acute isolation of the organ of Corti explants was performed as follows: Briefly, the mice were anesthetized with 1.2% pentobarbital sodium (60 mg/kg) and decapitated; the head of mice were collected and placed into a sterile Petri dish on ice. The temporal bone containing the inner ear was isolated from the skull using dissecting scissors and placed immediately into a fresh Petri dish containing an ice-cold cutting solution. Subsequently, the inner ears were isolated from the temporal bone to remove the Corti organ from the inner ear with forceps. Then the overlying tectorial membrane was removed with forceps. The organ of Corti explants was positioned under the pin of the coverslip for the electrophysiology clamp-patch experiment.

### Electrophysiology

Ionic currents were recorded from *X. laevis* oocytes under a standard excised inside-out patch clamp with Axon Digidata 1550 acquisition system (Axon Instruments) and Model 2400 patch clamp amplifier (A-M system). The pipette solution contains (in mM): 140 KMES (methanesulfonate), 20 KOH, 10 Hepes, 2 MgCl_2_, pH 7.0. The basal internal solution contains (in mM): 160 KOH, 140 MES, 20 Hepes, pH 7.5. The 0 μM Ca^2+^ solution was buffered by 5 mM EGTA. The 1 μM Ca^2+^ internal concentration was titrated by 5 mM EGTA and Ca (Mes)_2_ solution. The 4 μM and 10 μM Ca^2+^ solutions were titrated by 5 mM N-(2-hydroxyethyl)ethylenediamine-N,N′,N′-triacetic acid (HEDTA). The 100 μM Ca^2+^ internal solution was titrated by 0.1 ml 100 mM Ca (Mes)_2_ to total 500 ml internal solution. The internal solution was perfused to the cytoplasmic side of the patch. To obtain the GV curves, the currents were elicited by voltage pulses from −240 mV to +280 mV (40 ms) with 20 mV increments. The tail currents were measured and used to calculate the GVs of the BK variants according to procedures in previously published literature (49). The mechanical force was applied by stretching the cell membrane with the machine pneumatic transducer tester DPM1B (Fluke Biomedical). To avoid series resistance error, patches with maximal current under 10 nA were used. The linear leak current components were subtracted from the tail currents based on a linear slope calculated using the currents from −240 mV to −180 mV in each patch.

For BK current recording, organ of Corti explants was isolated from 3 to 8-week male and female mice and maintained in a standard physiological solution containing (in mM): 155 NaCl, 5.8 KCl, 0.9 MgCl_2_, 1.3 CaCl_2_, 0.7 NaH_2_PO_4_, 10 Hepes, 5.6 D-Glucose, 1 μM XE-991, 5 4-AP, pH adjusted to 7.4 with NaOH, osmolarity at 290 to 310 mOsm. The solution contained 1 μM XE-991 to inhibit KCNQ potassium channel current and 5 mM 4-action potential was used to inhibit IKs. The patch clamp pipettes had a resistance of 1.5 to 3 MΩ with an internal solution containing (in mM): 150 KCl, 3.5 MgCl_2_, 0.1 CaCl_2_, 5 EGTA, 2.5 Na_2_ATP, 5 HEPES, 15 4-AP, pH adjusted to 7.4 with NaOH, osmolarity at 290 to 310 mOsm. The IHCs in the apical-middle part of the cochleae were recorded. The BK channel currents were also identified by using the recorded total currents subtracted currents recorded with 200 nM IBTX in the extracellular solution. Experiments were processed at normal room temperature (22–25 °C).

### Western blot

Western blot analysis was performed referring to our previous studies (50). Briefly, total proteins (30 μg/sample) were extracted from the homogenized cochlea of WT mice or the KCNMA1 CKO mice at 8 to 12 weeks old. Protein concentration and yield were determined using a bicinchoninic acid protein assay kit (Beyotime). Total protein was electrophoresed and transferred by SDS-PAGE using 10% (w/v) acrylamide gels and blotted onto poly (vinylidene fluoride) membranes (Immobilon-P). Protein blots were blocked with 5% nonfat milk in tris buffered saline with tween 20 and incubated at 4 °C overnight with the primary KCa1.1 antibody (1:1000, Alomone Labs, APC-151), Beta-actin antibody (1:2000, Proteintech, 66009-1-lg). After washing, the poly (vinylidene fluoride) membranes were then washed and incubated with the secondary antibody (1:2000, Beyotime, A0208/A0216) with horseradish peroxidase-label for another 1 h at room temperature. The membranes were washed again in tris buffered saline with tween 20, developed with a clear Western enhanced chemiluminescence substrate (Beyotime), and imaged with an Alliance Q9 Advanced imaging system. Band intensities were determined using the ImageJ software (https://imagej.net/ij/). Beta-actin was used as a reference protein.

### Whole-mount immunofluorescence staining of mouse cochlea

Mice (C57BL/6, 8–12 weeks) were fully anesthetized with 1% pentobarbital (0.25 ml/25 g, intraperitoneal injection) and sacrificed by cervical dislocation. Cochleae were harvested and then fixed for 2 h in 4% paraformaldehyde at room temperature. Organs of Corti were decalcified in 10% EDTA for 24 h on a shaker at 4 °C and washed with PBS. After dissection of the organ of Corti from the cochlea, samples were treated with blocking solution (10% Goat Serum in PBS with 1% Triton X-100) for 1 h at room temperature. The organs of Corti were incubated with the primary antibodies [Anti-SAKCA antibody (a STREX-specific antibody was raised in rabbits using STREX-exon as antigen, 1:200,), and Myosin VIIa antibody (1:100) ]. After washing, the organs of Corti were incubated with the secondary antibodies [Alexa Fluor 568 goat anti-mouse (1:200); Alexa Fluor 488 goat anti-rabbit (1:200)] for 1 h at 37 °C. Finally, the organs of the Corti were stained with 4′,6-diamidino-2-phenylindole. The confocal images were performed using a Zeiss LSM 880 Confocal Microscope. The antibodies used were as follows: Goat anti-Mouse IgG H&L (Alexa Fluor 568): Abcam, ab175473; Goat anti-rabbit IgG H&L (Alexa Fluor 488): Abcam, ab150077; Anti-STREX Antibody (Sangon Biotech); and Myosin VIIa Antibody (Santa Cruz Biotechnology; Cat#: sc-74516).

### Data analysis

Electrophysiology data acquisition and analysis were carried out using Clampfit (Molecular Devices) software (https://www.moleculardevices.com/), and the statistical analysis was conducted with Prism 7.0 software (GraphPad, https://www.graphpad.com/) with the appropriate method illustrated in Figure legends. Data in all figures are presented as mean ± standard error (SE). Statistical significance was evaluated by a two-sample *t* test and *p* ≤ 0.05 was considered significant. GV curves were generally plotted using conductance calculated from macroscopic tail currents. The GV relations of the different channels were fitted with the Boltzmann equation:

$$G\;/\;G_{\max}=1\;/\;\left(1+\exp\left(-\text{ze}\;\left(V-V_{1/2}\right)\;/\;\text{kT}\right)\right)$$

where z is the number of equivalent gating charges, V_1/2_ is the voltage for the channel in half-maximal activation, e is the elementary charge, k is Boltzmann’s constant, and T is the absolute temperature. Each GV curve was obtained from 4 to 17 patches, and error bars represent the SEMs.

## Data availability

All data are contained within the article and Supporting information.

## Supporting information

This article contains supporting information Fig. S1–S4.

## Conflict of interest

The authors declare that they have no conflicts of interests with the contents of this article.

## Figures and Tables

**WB-KCNMA:**
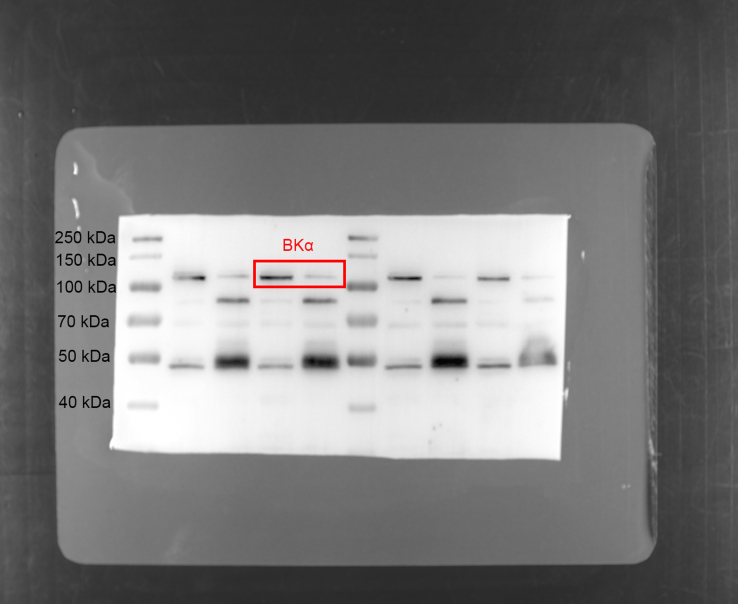


**WB-actin:**