# Supplementary material for: The mechanism of Ca2+-independent activation of BKCa channels in mouse inner hair cells and the crucial role of the BK channels in auditory perception
Source: J Biol Chem. 2024 Nov 7;301(1):107970. doi: 10.1016/j.jbc.2024.107970 (PMC11758846; doi:10.1016/j.jbc.2024.107970)
Supplement: Supplemental Figure Legend [file mmc1.docx]

**The mechanism of Ca^2+^ independent activation of BKCa channels in mouse inner hair cells and the crucial role of the BK channels in auditory perception**

Zhong-Shan Shen^1,2,3*^, Jun Gan^1,2,3*^, Bing Xu^4,5*^, Ya-Lin Chen^4,5^, Fei-Fei Zhang^1,2,3^, Jun-Wei Ji^1,2,3^, Dan-Hua Chen^1,2,3^, Yuehua Qiao^4,5^, Qiong-Yao Tang^1,2,3^, Zhe Zhang^1,2,3^

^1^Jiangsu Province Key Laboratory of Anesthesiology, Xuzhou Medical University, Xuzhou, Jiangsu Province 221004, China

^2^Jiangsu Province Key Laboratory of Anesthesia and Analgesia Application Technology, Xuzhou

Medical University, Xuzhou, Jiangsu Province, China

^3^NMPA Key Laboratory for Research and Evaluation of Narcotic and Psychotropic Drugs, Xuzhou Medical University, Xuzhou, Jiangsu Province, China

^4^ Otorhinolaryngology Department, The Affiliated Hospital of Xuzhou Medical University, Xuzhou, Jiangsu Province, China

^5^Auditory Engineering Laboratory of Jiangsu Province, Xuzhou Medical University, Xuzhou, Jiangsu Province, China

**Correspondence to:** [**oto8588@163.com**](mailto:oto8588@163.com)**,** [**Qiongyaotang@hotmail.com**](mailto:Qiongyaotang@hotmail.com)**, or** [Zhangzhe70@xzhmu.edu.cn](mailto:Zhangzhe70@xzhmu.edu.cn)

* These authors contribute equally to this manuscript

**Supporting Information contains Supplementary Figure 1 – 4.**

**Supplementary Figure 1. Effects of STREX-1 and STREX-1-e9alt channels on mechanical sensitivity. A – C.** Currents were activated with the indicated voltage protocol from -160 mV to +260 mV. Representative traces of recorded STREX-1 channel currents at 0 μM [Ca^2+^]_i_ in response to the membrane stretch at 0 mmHg (**A**), 40 mmHg (**B**), and 60 mmHg (**C**), respectively. **D – E.** Mean G-V relationship (D) and V_h_ (**E**) of STREX-1 channel with a stretch at 0 mmHg, 40 mmHg, and 60 mmHg in 0 μM, 10 μM, and 100 μM [Ca^2+^]_i,_ respectively (**D**). **F – H.** Currents were activated with the indicated voltage protocol from -200 mV to +180 mV. Representative current traces of STREX-1-e9alt channel in 0 μM [Ca^2+^]_i_ elicited by stretch at 0 mmHg (**F**), 40 mmHg (**G**), and 60 mmHg (**H**), respectively. **I – J.** Mean G-V relationship (**I**) and V_h_ (**J**) of STREX-1-e9alt channel with a stretch at 0 mmHg, 40 mmHg, and 60 mmHg in 0 μM, 10 μM, and 100 μM [Ca^2+^]_i_, respectively. **K.** ΔV_h_ between 0 mmHg and 40 mmHg or 60 mmHg of STREX-1 channel in 0 μM, 10 μM, and 100 μM [Ca^2+^]_i_, respectively. At each [Ca^2+^]_i_, ΔV_h_= V_h(STREX-1, at 0 mmHg)_ – V_h (STREX-1, at 40 mmHg)_ or ΔV_h_ = V_h (STREX-1, at 0 mmHg)_ – V_h (STREX-1, at 60 mmHg)_. **P* < 0.05. The data are presented as mean ± S.E., averaged from n = 4 - 9 patches. **L.** ΔV_h_ between 0 mmHg and 40 mmHg or 60 mmHg stretch for STREX-1-e9alt channel in 0 μM, 10 μM, and 100 μM [Ca^2+^]_i_, respectively. In each [Ca^2+^]_i_, ΔV_h_ = V_h (STREX-1-e9alt, at 0 mmHg)_ – V_h (STREX-1- e9alt, at 40 mmHg)_ or ΔV_h_ = V_h (STREX-1- e9alt, at 0 mmHg)_ – V_h (STREX-1- e9alt, at 60 mmHg)_. The data are presented as mean ± S.E., averaged from n = 4 patches. Details in Table I.

**Supplementary Figure 2. Effects of STREX-1 and STREX-1-e9alt channels on mechanical sensitivity under LRRC52 subunit regulation.**

**A – C.** Sample currents were generated with the indicated voltage protocol from -200 mV to +180 mV. Representative current traces of STREX-1 + LRRC52 in 0 μM Ca^2+^ elicited by stretch at 0 mmHg (**A**), 40 mmHg (**B**), and 60 mmHg (**C**) respectively. **D – E.** G-V relationship (**D**) and V_h_ (**E**) of STREX-1 + LRRC52 channel with a stretch at 0 mmHg, 40 mmHg, and 60 mmHg in 0 μM, 10 μM, and 100 μM [Ca^2+^]_i_, respectively. **F – H.** Representative current traces of STREX-1- e9alt + LRRC52 in 0 μM Ca^2+^ elicited by stretch at 0 mmHg (**F**), 40 mmHg (**G**), and 60 mmHg (**H**) respectively. **I – J.** Mean G-V relationship (**I**) and V_h_ (**J**) of STREX-1- e9alt + LRRC52 channel with a stretch at 0 mmHg, 40 mmHg, and 60 mmHg in 0 μM, 10 μM, and 100 μM [Ca^2+^]_i_, respectively. **K.** ΔV_h_ between 0 mmHg to 40 mmHg or 60 mmHg for STREX-1 + LRRC52 channel in 0 μM, 10 μM, and 100 μM [Ca^2+^]_i_, respectively. At each [Ca^2+^]_i_, ΔV_h_ = V_h (STREX-1 + LRRC52, at 0 mmHg)_ – V_h (STREX-1 + LRRC52, at 40 mmHg)_ or ΔV_h_ = V_h (STREX-1 + LRRC52, at 0 mmHg)_ – V_h (STREX-1 + LRRC52, at 60 mmHg)_. ***P* < 0.01. The data are presented as mean ± S.E., averaged from n = 5 patches. **L.** ΔV_h_ shift between 0 mmHg to 40 mmHg or 60 mmHg for STREX-1- e9alt + LRRC52 channel in 0 μM, 10 μM, and 100 μM [Ca^2+^]_i_, respectively. At each [Ca^2+^]_i_, ΔV_h_ = V_h (STREX-1-_ _e9alt + LRRC52, at 0 mmHg)_ – V_h (STREX-1- e9alt + LRRC52, at 40 mmHg)_ or ΔV_h_ = V_h (STREX-1- e9alt + LRRC52, at 0 mmHg)_ – V_h (STREX-1- e9alt + LRRC52, at 60 mmHg)_. **P* < 0.05. The data are presented as mean ± S.E, averaged from n = 5 patches. Details in Table I.

**Supplementary Figure 3. Outward currents in WT IHCs are inhibited by 200 nM IBTX. A.** Representative traces show the effect of injected voltage (-100 ~ +50 mV, step +5 mV) induced outward currents in the IHC from the WT mice. **B.** The inhibition of outward currents by 200 nM extracellular IBTX. **C.** Subtraction of the outward currents recorded in the presence of IBTX from the total outward currents recorded in the control solution reveals the BK current in IHCs from WT mice. **D.** Normalized conductance-voltage plots obtained from the analysis of steady-state with Boltazman equation fit. V_h_ = -18.4 ± 1.46 mV, mean values from 6 WT IHC cells.

**Supplementary Figure 4. The designed strategy of KCNMA1 CKO mice and genotype samples. A.** Schematic diagram of FloxP flanked KCNMA1 conditional knockout strategy by using CRISPR/Cas9 technology. **B.** PCR-based test of KCNMA1-flox mice genotype. The WT and homozygous alleles generated 310 bp and 415 bp DNA bands in PCR, respectively.
