# Supplementary figures and images for: The mechanism of Ca2+-independent activation of BKCa channels in mouse inner hair cells and the crucial role of the BK channels in auditory perception
Source: J Biol Chem. 2024 Nov 7;301(1):107970. doi: 10.1016/j.jbc.2024.107970 (PMC11758846; doi:10.1016/j.jbc.2024.107970)

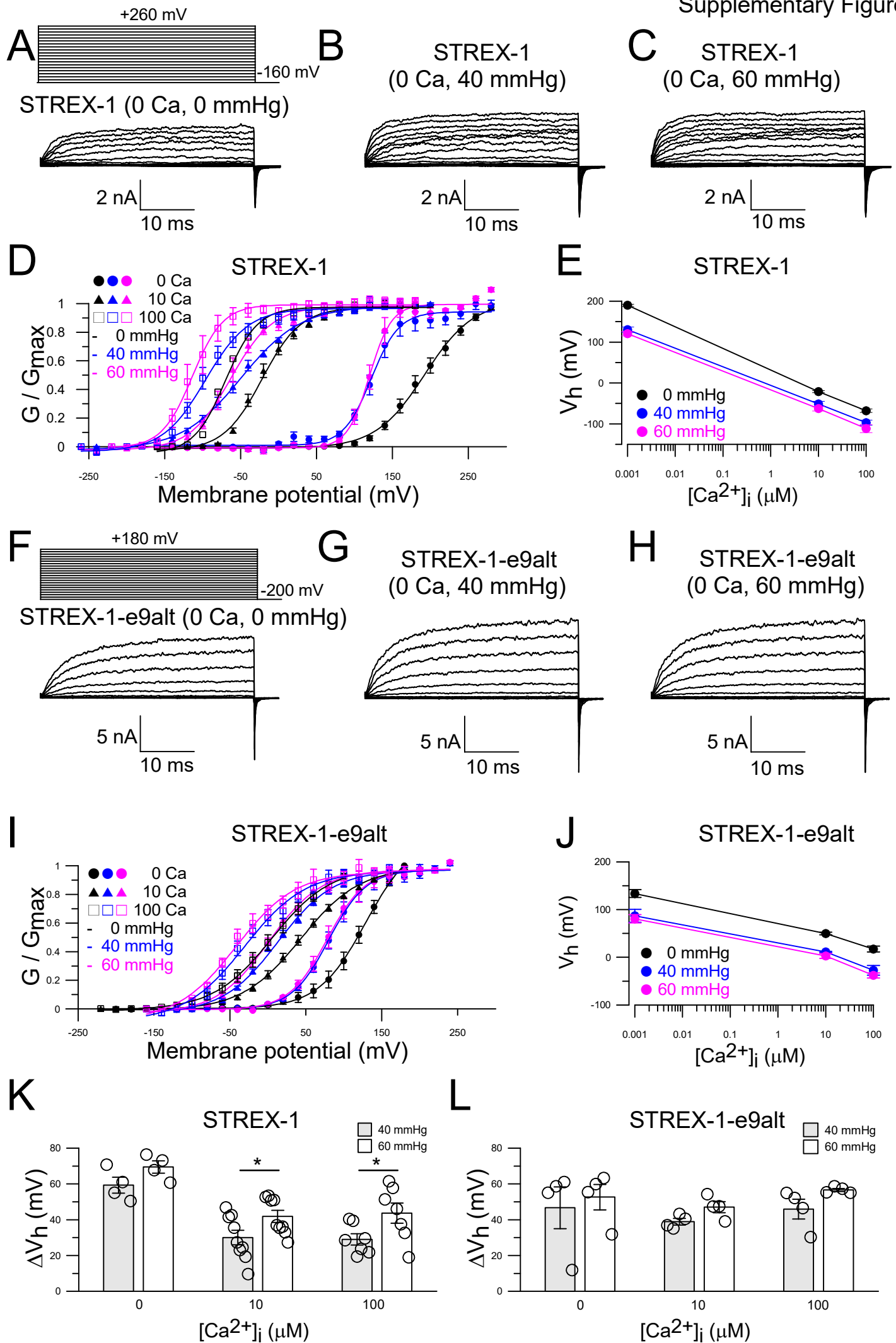

Supplement: Supplemental Figure S1 [file mmc2.pdf]

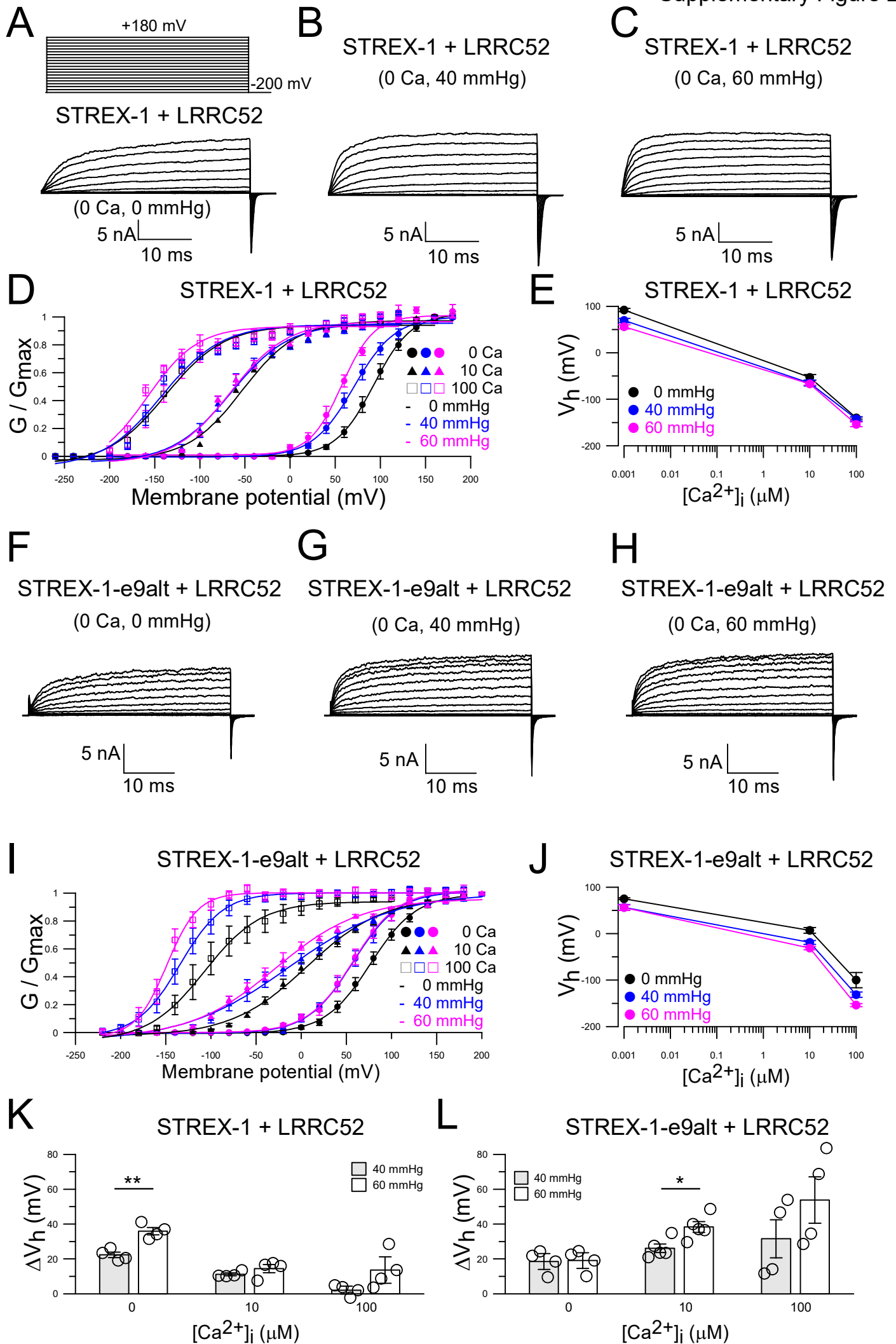

Supplement: Supplemental Figure S2 [file mmc3.pdf]

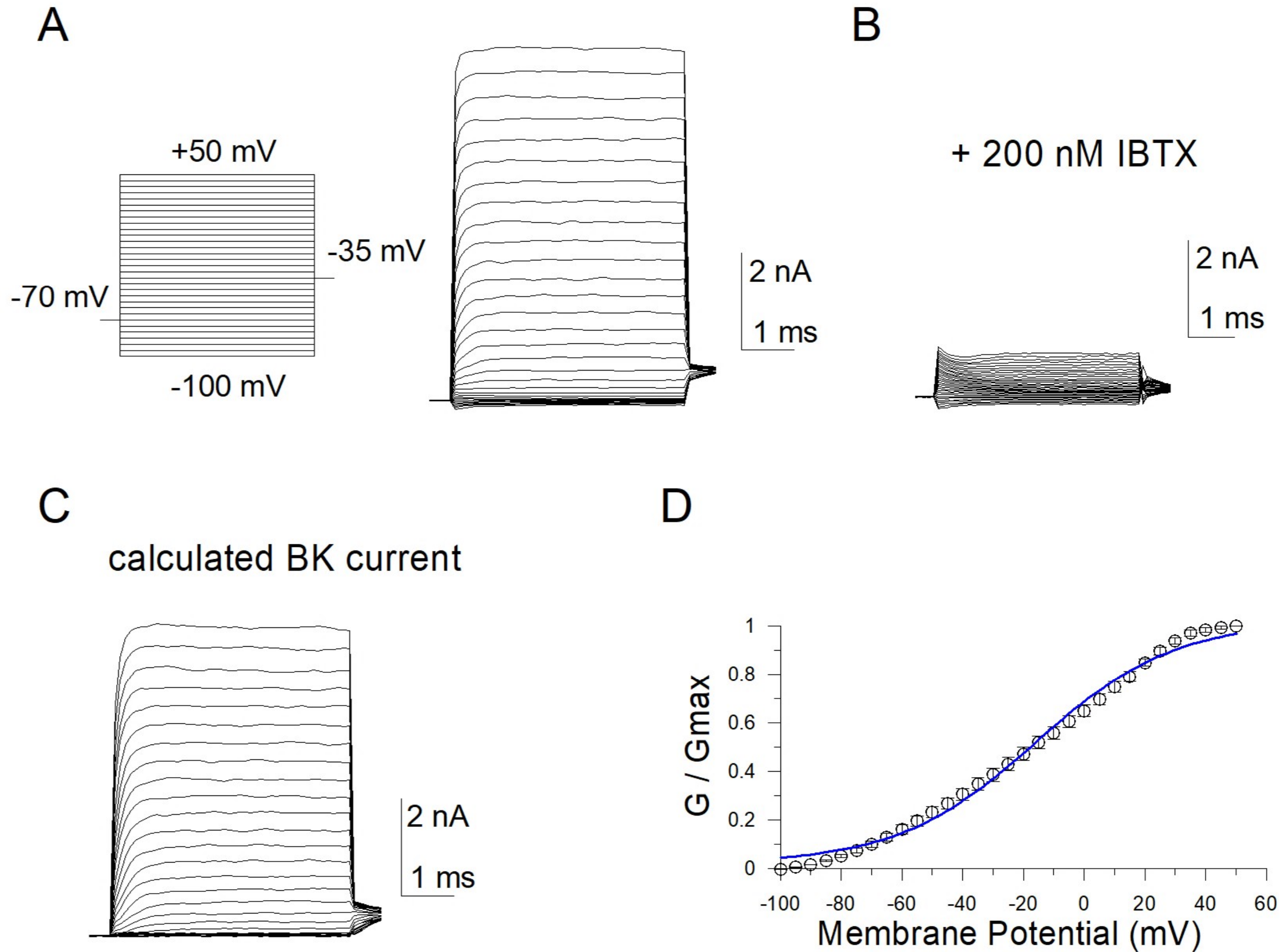

Supplement: Supplemental Figure S3 [file mmc4.pdf]
