# Supplementary material for: The mechanism of Ca2+-independent activation of BKCa channels in mouse inner hair cells and the crucial role of the BK channels in auditory perception
Source: J Biol Chem. 2024 Nov 7;301(1):107970. doi: 10.1016/j.jbc.2024.107970 (PMC11758846; doi:10.1016/j.jbc.2024.107970)

Supplementary Figure 4

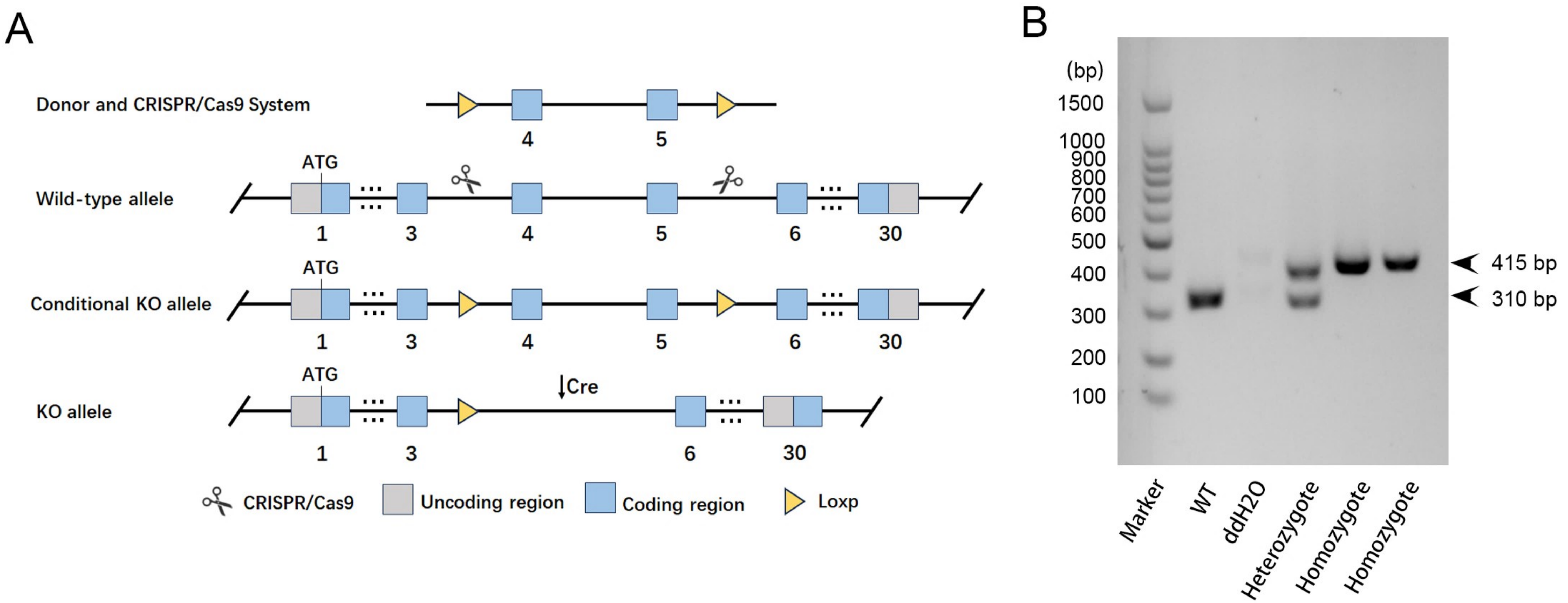

Supplement: Supplemental Figure S4 [file mmc5.pdf]
